# RAS-dependent RAF-MAPK hyperactivation by pathogenic RIT1 is a therapeutic target in Noonan syndrome–associated cardiac hypertrophy

**DOI:** 10.1126/sciadv.adf4766

**Published:** 2023-07-14

**Authors:** Antonio Cuevas-Navarro, Morgan Wagner, Richard Van, Monalisa Swain, Stephanie Mo, John Columbus, Madeline R. Allison, Alice Cheng, Simon Messing, Thomas J. Turbyville, Dhirendra K. Simanshu, Matthew J. Sale, Frank McCormick, Andrew G. Stephen, Pau Castel

**Affiliations:** ^1^ Helen Diller Family Comprehensive Cancer Center, University of California, San Francisco, San Francisco, CA 94158, USA.; ^2^ NCI RAS Initiative, Cancer Research Technology Program, Frederick National Laboratory for Cancer Research, Leidos Biomedical Research Inc., Frederick, MD 21702, USA.; ^3^ Department of Biochemistry and Molecular Pharmacology, New York University Grossman School of Medicine, New York, NY 10016, USA.

## Abstract

RIT1 is a RAS guanosine triphosphatase (GTPase) that regulates different aspects of signal transduction and is mutated in lung cancer, leukemia, and in the germline of individuals with Noonan syndrome. Pathogenic RIT1 proteins promote mitogen-activated protein kinase (MAPK) hyperactivation; however, this mechanism remains poorly understood. Here, we show that RAF kinases are direct effectors of membrane-bound mutant RIT1 necessary for MAPK activation. We identify critical residues in RIT1 that facilitate interaction with membrane lipids and show that these are necessary for association with RAF kinases and MAPK activation. Although mutant RIT1 binds to RAF kinases directly, it fails to activate MAPK signaling in the absence of classical RAS proteins. Consistent with aberrant RAF/MAPK activation as a driver of disease, we show that pathway inhibition alleviates cardiac hypertrophy in a mouse model of *RIT1* mutant Noonan syndrome. These data shed light on the function of pathogenic RIT1 and identify avenues for therapeutic intervention.

## Teaser

Electrostatic plasma membrane binding facilitates RIT1-mediated RAS-dependent RAF activation to promote pathogenic MAPK signaling.

## INTRODUCTION

The RAS family of small guanosine triphosphatases (GTPases) control a diverse network of signaling pathways essential for human development and adult tissue homeostasis (*1*, *2*). The activity of RAS GTPases is dictated by their nucleotide-loading state and is subject to various regulatory mechanisms that respond to extracellular stimuli and feedback signals (*2*). RAS GTPases bound to guanosine diphosphate (GDP) are considered inactive, while their guanosine triphosphate (GTP)–bound conformation promotes activation of downstream effectors. GTPase-activating proteins (GAPs), such as NF1 and RASA1/p120GAP, turn off RAS proteins by catalyzing GTP hydrolysis. Conversely, guanine nucleotide exchange factors (GEFs), such as SOS1/SOS2, activate RAS by promoting the exchange of GDP for GTP. When bound to GTP, the classical RAS proteins HRAS, NRAS, and KRAS (hereinafter referred to as RAS) recruit RAF kinases and other effector proteins to the plasma membrane (PM) to activate various downstream signaling cascades. Translocation of RAF to the PM, mediated by its high-affinity RAS-binding domain (RBD), is a crucial step in the activation of the RAF/MAPK kinase (MEK)/extracellular signal–regulated kinase (ERK) mitogen-activated protein kinase (MAPK) pathway (*3*, *4*). Hyperactivation of this critical RAS signaling axis is a hallmark of many cancers and the cause of a group of developmental syndromes collectively called RASopathies (*5*).

Recently, gain-of-function mutations in the RAS-related GTPase *RIT1* have emerged as drivers of human disease, including lung adenocarcinoma and myeloid malignancies (*6*–*8*). Germline *RIT1* variants cause Noonan syndrome (NS), a RASopathy characterized by craniofacial dysmorphism, short stature, and congenital heart disease (*9*–*11*). Patients with RIT1 NS make up approximately 5 to 10% of all NS cases and exhibit congenital cardiac defects at elevated frequencies (*9*, *12*). Recent murine models of RIT1 NS, independently developed by our group and others, present features that recapitulate clinical manifestations, including a shortened stature, craniofacial dysmorphism, and cardiac hypertrophy (*13*, *14*), providing an avenue for the evaluation of therapies against hypertrophic cardiomyopathy (HCM) and other cardiac defects closely associated with RIT1 NS in a preclinical setting.

RIT1 is expressed in many tissues and, similar to other RAS family GTPases, associates with the inner leaflet of the PM through a unique C-terminal hypervariable region (HVR). The G domain of RIT1 shares 51% sequence identity with RAS and thus can potentially associate with an overlapping set of effector proteins, including the RAF kinases. However, regulation of the RIT1 GTPase cycle remains elusive, with cognate RIT1 GAPs and GEFs yet to be identified. A high fraction of cellular RIT1 is bound to GTP, even in the absence of mitogenic signals, suggesting that RIT1 activity may rely on its intrinsic nucleotide exchange and hydrolase activity and/or alternative regulatory mechanisms (*13*). One such mechanism involves the NS-associated protein leucine-zipper-like transcription regulator 1 (LZTR1), which functions as a conserved substrate receptor for Cullin3 E3 ubiquitin ligase complexes (CRL3^LZTR1^) to promote the ubiquitination and proteasomal degradation of GDP-bound RIT1 (*15*). NS pathogenic *RIT1* and *LZTR1* variants disrupt RIT1-LZTR1 binding, resulting in the accumulation of RIT1 protein (*13*, *16*) and enhanced MAPK signaling; however, it remains unclear how evasion of LZTR1-mediated degradation resulting in RIT1 protein overabundance contributes to MAPK pathway activation.

Here, we use biophysical, biochemical, and cell biological approaches to interrogate RIT1 activation of RAF kinases. We show that different biochemical properties of the RIT1 HVR contribute to its association with the PM and enable RAF binding and MAPK activation. RIT1 exhibits preferential binding to RAF1 (also known as CRAF) and engages with an overlapping set of RBD residues associated with RAS binding, albeit with a weaker affinity. Furthermore, we demonstrate that the absence of RAS proteins and RAS:RAF binding limits the ability of pathogenic RIT1 to hyperactivate the MAPK pathway and that pharmacological MAPK inhibition ameliorates cardiac tissue overgrowth associated with aberrant RIT1 signaling in a RIT1 NS mouse model.

## RESULTS

### RIT1 oncoproteins activate RAF kinase

**Fig. 1.**
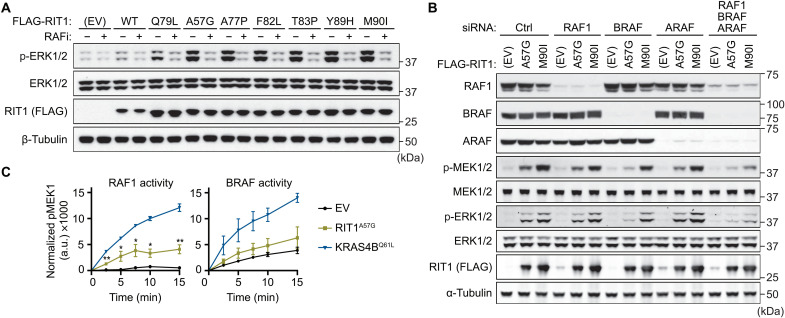
RIT1 oncoproteins activate ERK signaling via RAF kinase. (**A**) Immunoblot analysis of indicated proteins from HEK293T cells transiently transfected with indicated FLAG-tagged RIT1 constructs or an EV control and treated with 10 μM LY3009120 (RAFi) or DMSO vehicle control for 1 hour. One of two independent experiments is shown. (**B**) Immunoblot analysis of indicated proteins from HEK293T cells transiently transfected with indicated siRNA and FLAG-tagged RIT1 constructs or EV control. One of two independent experiments is shown. (**C**) In vitro MEK1 phosphorylation (Ser^218^/Ser^222^) by RAF1 or BRAF protein isolated from HEK293T cells coexpressing RIT1^A57G^, KRAS^Q61L^, or an EV control. a.u., arbitrary units. Data points indicate the means ± SEM of four (RAF1) or three (BRAF) biological replicates (independently isolated RAF protein samples). p, phospho.

Although expression of pathogenic RIT1 variants has been widely demonstrated to promote MEK/ERK pathway activation, the mechanism by which RIT1 activates MAPK signaling remains unclear (*6*, *10*, *11*, *13*). RIT1 associates with RAF kinases in a nucleotide-dependent manner (*17*), a defining feature of GTPase effector proteins, suggesting a direct link between RIT1 and MEK/ERK activation. To assess whether RIT1 oncoproteins activate MAPK signaling in a RAF-dependent manner, we analyzed ERK phosphorylation in cells expressing either RIT1 wild-type (WT) or different RIT1 mutants following treatment with a third-generation small-molecule pan-RAF inhibitor (*18*). Pharmacological RAF inhibition abolished MAPK activation induced by ectopic RIT1 expression, including RIT1^Q79L^ (a constitutively active mutant analogous to RAS^Q61L^), and a panel of NS and cancer-associated variants with variable levels of GTP-loading in cells (Fig. 1A) (*13*). Furthermore, knockdown of the three RAF paralogs (ARAF, BRAF, and RAF1) by RNA interference (RNAi) decreased mutant RIT1-induced ERK phosphorylation, highlighting a dependence on RAF kinases (Fig. 1B). To determine whether mutant RIT1 activates RAF, we measured the activity of RAF proteins isolated from mammalian cells coexpressing RIT1^A57G^, the most common RIT1 NS variant, or an empty vector (EV) control (Fig. 1C and fig. S1). BRAF exhibited high basal activity in vitro as previously observed (*19*), potentially due to constitutive phosphorylation of its activation loop (*20*), but was largely refractory to RIT1^A57G^, but not KRAS^Q61L^, coexpression. In contrast, RIT1^A57G^ substantially enhanced RAF1 kinase activity, albeit to a lesser extent than KRAS^Q61L^. Together, these data suggest that mutant RIT1 activates RAF kinases in cells and that the RAF-MEK-ERK cascade is a putative direct effector pathway of mutant RIT1.

### RIT1 association with the PM requires charge complementarity

**Fig. 2.**
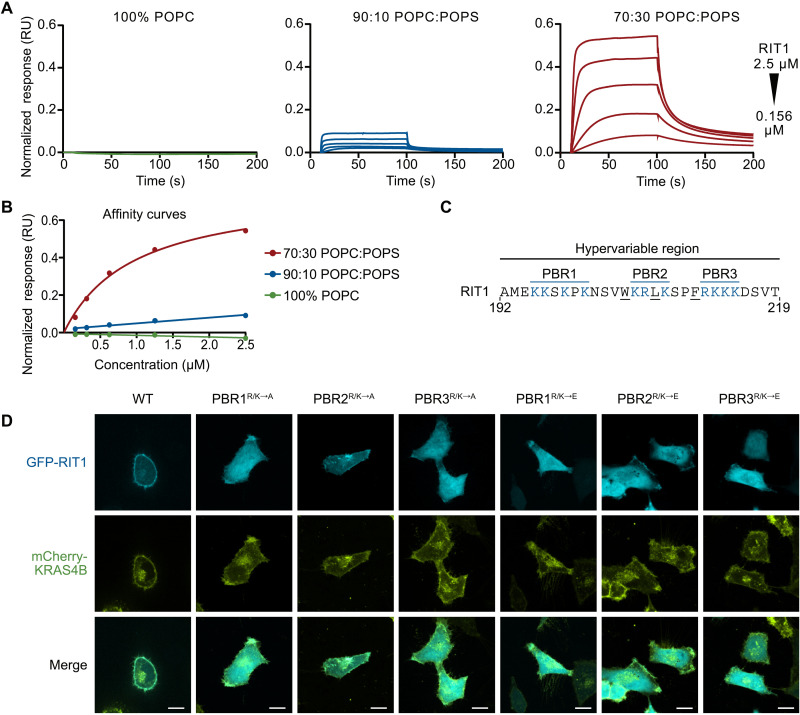
RIT1 associates with the PM through its HVR. (**A**) SPR analysis with increasing concentrations of POPS-containing liposomes showing RIT1 (amino acids 17 to 219) association with negatively charged lipids. (**B**) SPR affinity curves showing relative binding affinity of RIT1 to lipid constructs. (**C**) RIT1 C-terminal amino acid sequence. PBRs are colored blue. (**D**) Live-cell confocal images of HeLa cells transiently transfected with indicated GFP-RIT1 constructs. Stable expression of mCherry-KRAS4B was used as a PM marker. Representative images from one of three independent experiments (*n* = 3). Scale bars, 15 μm. RU, response units.

Given the crucial role played by the PM in the activation of RAF kinases, we sought to investigate the association of RIT1 with the inner leaflet of the PM. Unlike classical RAS proteins, the RIT1 HVR lacks prenylation motifs, indicating that RIT1 engages with the PM in a unique way. To investigate this interaction, we used surface plasmon resonance (SPR) to measure the association of RIT1 with liposomes of various charge ratios (Fig. 2A). Although RIT1 showed no binding to neutral liposomes, we observed an enhanced binding response as the negative charge in the liposome was increased via the inclusion of 16:0 to 18:1 1-palmitoyl-2-oleoyl-*sn*-glycero-3-phospho-l-serine (POPS) (Fig. 2B). We confirmed that this interaction was strictly mediated by the HVR because binding with negatively charged liposomes was not observed after deletion of this domain (fig. S2A). The association of RIT1 with anionic lipid–containing liposomes was quantitated by calculating the partition coefficient (fig. S2B) (*21*). Increasing the concentration of POPS in the liposomes from 10 to 30% increased the partition coefficient by 40-fold. Consistent with our data, molecular dynamic simulations have found that the RIT1 HVR experiences a substantially longer residence time on POPS-containing bilayers compared to uncharged bilayers (*22*).

The RIT1 HVR has an isoelectric point of 10.6 and contains three polybasic regions (PBRs) separated by noncharged amino acids (Fig. 2C). To better characterize the contribution of these PBRs, we assessed the PM association of green fluorescent protein (GFP)–tagged RIT1 with individual PBRs that had their charge neutralized (R/K → A) or reversed (R/K → E) (Fig. 2D). As expected, charge reversal and neutralization of PBR1, PBR3, and, to a lesser degree, PBR2 disrupted the typical distribution of RIT1 at the cellular periphery, suggesting that all three PBRs are essential for membrane association in cells. PBR2 contains fewer basic residues than PBR1 and PBR3, and thus, the contribution to membrane association provided by each PBR may be directly correlated to their overall charge contribution. In addition, charge neutralization of a single basic residue within PBR2 or PBR3 (Arg^206^ and Arg^212^, respectively) was insufficient to disrupt membrane association (fig. S2C). Using a GFP-RIT1 C-terminal peptide fusion construct, Heo *et al.* (*23*) demonstrated that hydrophobic side chains of the RIT1 HVR may also regulate RIT1-PM association. Therefore, we individually mutated five hydrophobic HVR residues in a full-length GFP-RIT1 construct. Of these, alanine substitution of the three largest side chains disrupted PM targeting (fig. S2C). Collectively, these data indicate that charge complementarity plays a substantial role in the association of RIT1 with the inner leaflet of the PM and that this interaction receives a contribution from the hydrophobic residues interspersed between the PBRs. Notably, common pathogenic RIT1 mutations did not affect PM association (fig. S2D).

### Characterization of RIT1-RAF1 interaction identifies critical residues

**Fig. 3.**
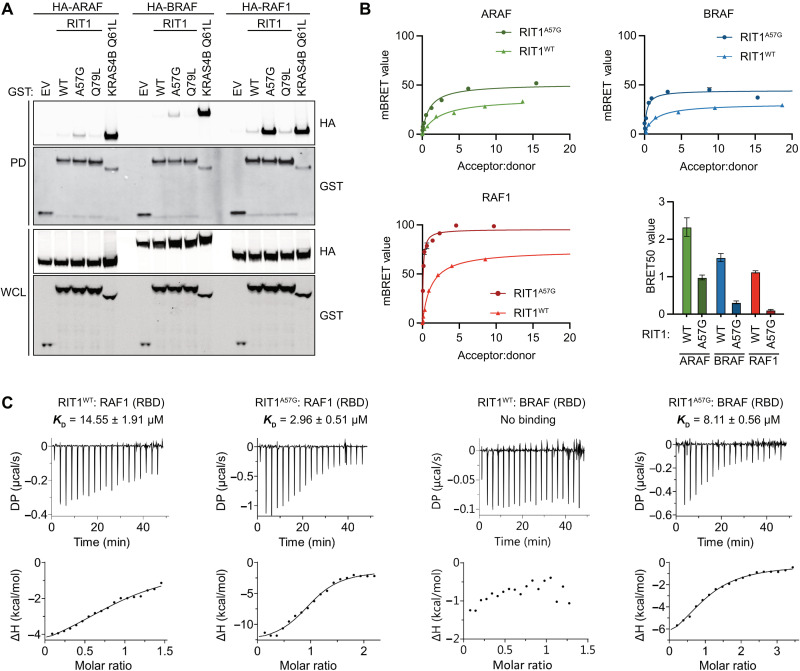
RIT1 exhibits preferential binding to RAF1. (**A**) Immunoblot analysis of indicated proteins precipitated by GST pull-down assay from HEK293T cell lysates expressing indicated constructs. DNA amounts for WT and mutant RIT1 were adjusted to normalize for protein expression. EV, empty vector; WCL, whole-cell lysate; PD, pulldown. (**B**) BRET curves show the relative binding affinities of mVenus-RIT1 (acceptor) and RAF-nanoLuc (donor) proteins. Representative BRET curves from three independent experiments are shown. The histogram demonstrates the mean BRET_50_ values ± SD of three independent experiments. (**C**) ITC measurements of recombinant RIT1:RAF(RBD) binding affinities. *K*_D_ values represent an average of three independent experiments (*n* = 3).

The HVRs of classical RAS proteins contribute to distinct binding preferences with the different RAF family members (*24*). Given RIT1’s unique HVR, we sought to determine whether RIT1 also exhibits preferential RAF paralog binding. Notably, binding of the three RAF paralogs to WT RIT1 was nearly undetectable in pull-down assays and was markedly weaker than their affinity to KRAS4B (Fig. 3A). However, in contrast to prior observations (*17*), a notable preference for RAF1 was observed when using the pathogenic RIT1^A57G^ variant. Thus, to better assess these interactions, we developed a quantitative bioluminescence resonance energy transfer (BRET) assay to quantitate the association of RIT1 with RAF kinases in cells (fig. S3, A and B). WT RIT1 had higher maximal BRET value (BRET_max_) and lower Acceptor:donor ratio at half maximal BRET value (BRET_50_) values for RAF1 compared to BRAF and ARAF, indicating a preference for the RAF1 paralog in intact cells (Fig. 3B and fig. S3C). On the basis of the calculated BRET_50_ values, RIT1 binds preferentially to RAF1 over BRAF and ARAF. RIT1^A57G^ bound to RAF1 around 10-fold tighter than WT RIT1 and showed the same binding preferences as the WT protein: RAF1 > BRAF > ARAF. To investigate this interaction further, we used isothermal titration calorimetry (ITC) to measure the binding affinity between recombinant RAF1-RBD or BRAF-RBD and WT RIT1 or RIT1^A57G^ bound to GMPPNP, a nonhydrolyzable GTP analog (Fig. 3C). In these experiments, RIT1^A57G^ [dissociation constant (*K*_D_) = 2.96 μM] bound approximately five times more tightly to RAF1-RBD compared with WT (*K*_D_ = 14.55 μM). Consistent with our BRET measurements, RIT1^A57G^ interaction with BRAF-RBD was weaker than RAF1-RBD. However, no measurable binding was observed between WT RIT1 and BRAF-RBD, presumably because the binding interaction was below the detection limit for ITC. Cumulatively, these data suggest that RIT1 preferentially interacts with RAF1 over the other RAF paralogs.

**Fig. 4.**
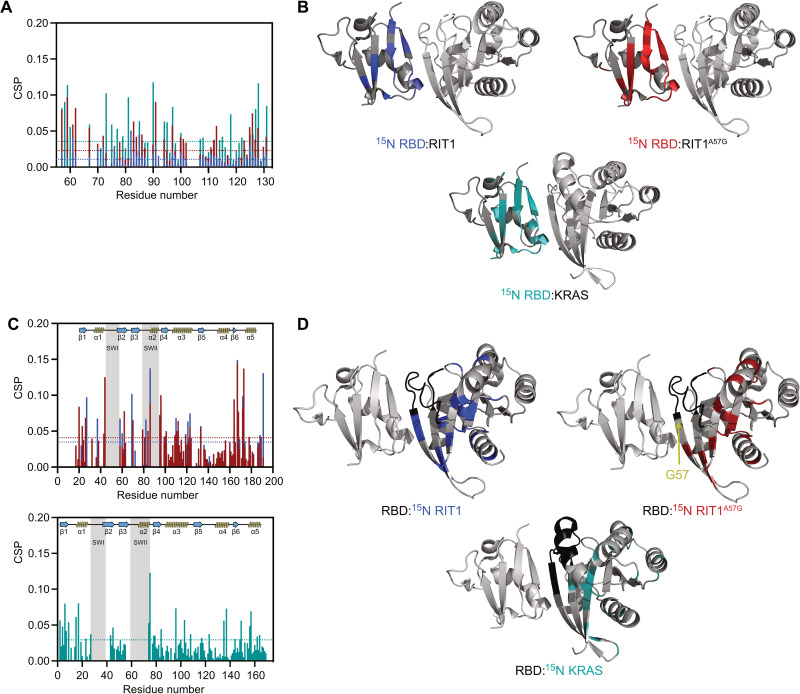
Characterization of RIT1-RAF RBD interface by NMR. (**A**) CSP plots for ^15^N RAF1-RBD observable in complex with unlabeled WT RIT1 (blue), RIT1^A57G^ (red), and WT KRAS (cyan). Dashed lines represent 1.5σ. (**B**) CSP shown in (A) and broadened residues were mapped to the 3D-modeled RBD-RIT1 complex and RBD-KRAS structure [Protein Data Bank (PDB): 6VJJ]. (**C**) The top panel represents the CSP for ^15^N WT RIT1 (blue) and ^15^N RIT1^A57G^ (red) in complex with unlabeled RBD. The bottom panel represents CSP for ^15^N WT KRAS in complex with unlabeled RAF1-RBD. Dashed lines represent 1.5σ. (**D**) CSP shown in (C) and broadened residues were mapped to the 3D structures as in (B).

To understand how RAF binding differs between RIT1 and RAS, we used solution nuclear magnetic resonance (NMR) spectroscopy to identify broadened and induced chemical shifts upon RBD binding (table S1 and fig. S4). An overlay of the RAF1-RBD chemical shift perturbation (CSP) histograms produced by binding to WT RIT1, RIT1^A57G^, or KRAS revealed an overlapping set of perturbed residues with minor variations (Fig. 4A). Notably, differences in binding affinities were evident by CSP analysis and were congruent with affinities measured in vitro and in cells (Fig. 3); specifically, WT RIT1 induced the smallest perturbation of resonances, followed by RIT1^A57G^, and then KRAS, which induced the largest perturbations. Perturbed residues were then mapped onto modeled structures of WT and A57G mutants of RIT1 in the active state and were compared with the previously solved crystal structure of the KRAS:RAF1-RBD complex (Fig. 4B) (*25*). This identified the putative RIT1:RBD interface and confirmed a shared binding site on RAF1-RBD. CSP analysis of RIT1 revealed residues (Met^37^, Ser^43^, and His^44^) at the N-terminal end of the switch I flexible loop, respectively, that were considerably perturbed in the A57G mutant but not in the WT form (Fig. 4, C and D; table S1; and fig. S4, D to F). These data suggest that the switch I of RIT1^A57G^ differentially engages RAF1-RBD, potentially providing the enhanced stability exhibited by the pathogenic variant.

**Fig. 5.**
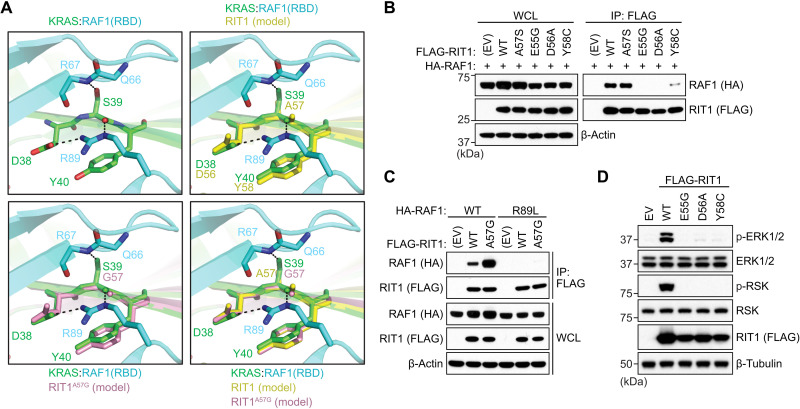
Analysis of the RIT1-RAF RBD structural interface. (**A**) Comparison of modeled structures of WT and A57G mutant of RIT1 with the crystal structure of KRAS:RAF1(RBD) complex (PDB: 6VJJ). Top left: Interaction formed by KRAS Ser^39^ (equivalent to Ala^57^ in RIT1) and neighboring residues Asp^38^ and Tyr^40^ with RAF1-RBD. KRAS and RAF1-RBD are colored green and cyan, respectively. Top right: Superposition of KRAS:RAF1(RBD) complex with the modeled structure of WT RIT1 (colored yellow) in the active state. Bottom left: Superposition of KRAS:RAF1(RBD) complex with the modeled structure of A57G mutant of RIT1 (colored pink) in the active state. Bottom right: Superposition of KRAS:RAF1(RBD) complex with the modeled structures of WT and A57G mutant of RIT1 in the active state. (**B** and **C**) Immunoblot analysis of indicated proteins immunoprecipitated from HEK293T cell lysates expressing indicated constructs. (**D**) Immunoblot analysis of indicated proteins from HEK293T cells transiently transfected with indicated FLAG-tagged RIT1 constructs or an EV. For (B) to (D), one of two independent experiments is shown.

To further interrogate how the A57G mutation in RIT1 enhances its interaction with RAF1, we undertook a comparative analysis of the modeled RIT1:RAF1-RBD structures with that of the solved KRAS:RAF1-RBD structure (Fig. 5A) (*25*). The RIT1 Ala^57^ residue located at the end of the switch I region is equivalent to Ser^39^ in KRAS. In the KRAS:RAF1-RBD complex structure, side-chain and main-chain atoms of Ser^39^ form hydrogen (H) bonds with Arg^67^ and Arg^89^ residues of RAF1. KRAS residues Asp^38^ and Tyr^40^, which surround amino acid Ser^39^, form key interactions with RAF1-RBD by forming salt bridge, H bond, and van der Waals interaction with RAF1 Arg^89^ and Thr^68^ residues (*25*). R89L mutation in RAF1 and mutations of Ser^39^ neighboring residues (E37G, D38A, and Y40C mutations) in RAS proteins have been shown to result in either complete or substantial loss of binding between KRAS and RAF1-RBD (*21*, *25*–*27*), suggesting that the interactions formed by Ser^39^ and residues around it in KRAS and Arg^89^ in RAF1 play a critical role in KRAS:RAF interaction. To assess whether the corresponding residues in RIT1 also form key contacts with the RAF1-RBD, we introduced analogous mutations (E55G, D56A, and Y58C), all of which resulted in a complete or notable loss of binding between RIT1 and RAF1 (Fig. 5B). Similarly, RAF1^R89L^ failed to bind WT RIT1 or RIT1^A57G^ (Fig. 5C). Notably, RIT1 mutants unable to bind RAF1 failed to activate MAPK signaling (Fig. 5D). Unlike the KRAS Ser^39^ residue, the Ala^57^ or Gly^57^ (A57G) residues in RIT1 cannot form an H bond with Arg^67^ as they lack a side-chain hydroxyl group (Fig. 5A); however, mutating Ala^57^ to serine was insufficient to promote tighter RAF1 binding (Fig. 5B), suggesting that the absence of the H bond with Arg^67^ alone does not explain the weaker affinity exhibited by RIT1. Glycine is fundamentally different from alanine and all other amino acids in that it lacks a side chain, which allows much larger rotational freedom of its main-chain torsion angles (*28*). In the β strand, glycine is often present at N-cap and C-cap positions, and the presence of glycine in the middle of the β strand has been shown to have a strong tendency to block β sheet continuation (*29*). Thus, unlike Ala^57^ in WT RIT1, the Gly^57^ residue in RIT1 has increased rotational space of main-chain torsion angles, imparting flexibility to the local peptide structure. This allows Gly^57^ and neighboring residues in switch I of RIT1 to undergo minor conformational changes that permit higher-affinity interactions with RAF1-RBD, as suggested by the additional perturbation and broadening of RAF1 interface residues Arg^67^ and Val^70^, respectively, similar to the conformational changes observed upon KRAS:RAF1-RBD complex formation (table S1). Thus, the A57G mutation likely enhances the RIT1:RAF1 interaction through increased flexibility in its main-chain torsion angles, which affect not only Gly^57^ but also neighboring residues, including switch I and flanking residues (Ser^43^ and His^44^) (Fig. 4C). Together, these play a crucial role in forming an enhanced interaction with RAF1-RBD.

### RIT1 membrane localization is required for RAF interaction

**Fig. 6.**
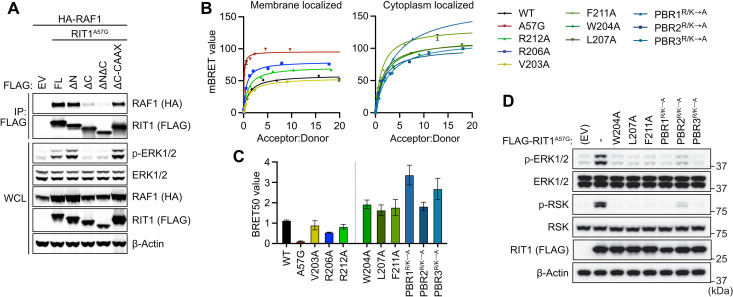
RIT1 HVR is required for RAF binding. (**A**) Immunoblot analysis of indicated proteins immunoprecipitated from HEK293T cell lysates expressing indicated constructs. EV, empty vector; ΔN, amino acid 1 to 18 deletion; ΔC, amino acid 192 to 219 deletion; WCL, whole-cell lysate. (**B**) BRET assays of indicated C-terminal HVR mutants associated with RAF1-nanLuc. One of three experiments is shown. (**C**) Histogram of BRET_50_ values indicating the relative binding affinities of RIT1 C-terminal mutants for RAF1-nanoLuc. Mean BRET_50_ values ± SD of three independent experiments. (**D**) Immunoblot analysis of indicated proteins from HEK293T cells transiently transfected with indicated FLAG-tagged RIT1 constructs or an EV. One of two independent experiments is shown.

RAS:RAF-RBD binding is insufficient for RAF activation in the absence of vicinal PM phospholipids, and thus, PM anchoring is a requisite for RAS-driven MAPK activation (*30*). Therefore, we reasoned that RIT1:RAF binding and activation may exhibit similar dependency on PM association. To assess the role of RIT1 membrane trafficking on RAF activation, we expressed N-terminal and C-terminal RIT1 deletion mutants and assessed binding by pull-down assay. As predicted, deletion of the RIT1 C terminus (192 to 219), but not its N terminus (1 to 18), disrupted membrane association, RAF1 binding, and MAPK activation (Fig. 6A and fig. S5A). When membrane association of the C-terminal deletion mutant was rescued by introducing a CAAX box motif, RIT1:RAF1 binding and MAPK activation were restored, indicating that PM localization of RIT1 is required for productive interaction with RAF1. Notably, when measuring relative RIT1 GTP loading in a RAF1-RBD pull-down assay, deletion of the RIT1 C terminus had no effect on GTP-dependent RBD binding that was abrogated by a dominant negative S35N mutation (*17*, *31*), equivalent to S17N in RAS, which destabilizes nucleotide binding (fig. S5B) (*32*). These data suggest that, while necessary for RIT1-mediated RAF activation, PM binding is dispensable for GTP loading.

Given the critical function of the HVR in membrane localization, we sought to quantitatively assess how its biochemical properties contribute to RIT1:RAF1 binding in cells. Point mutations that had no impact on membrane localization (fig. S2C), such as V203A, R206A, and R212A, bound RAF1 with a BRET_50_ comparable to WT RIT1 (Fig. 6, C and B). However, mutations that substantially decreased membrane localization produced much larger BRET_50_ values, indicating a weaker association with RAF1, and suppressed MAPK activation by RIT1^A57G^ (Fig. 6D). Charge-neutralizing mutations in PBR1 and PBR3 had the largest impact on RAF1 binding, consistent with their effect on RIT1 PM localization (Fig. 2D). Together, these findings suggest that while the RIT1 HVR does not directly participate in the activation of RAF, it is necessary for RIT1 localization to the PM where it can interact with RAF and activate the MAPK cascade.

### Mutant RIT1 activates RAF in a RAS-dependent manner

Given that RIT1 binds RAF directly, one can posit that RIT1, similar to RAS, may promote downstream MAPK signaling via direct recruitment and activation of RAF at the PM and that MAPK activation may be limited by RIT1’s weak affinity toward RAF (*13*). Despite an increased affinity toward RAF, RIT1^A57G^ exhibits comparable MAPK activation relative to other pathogenic variants with an affinity toward RAF that is indistinguishable from WT RIT1, including the oncogenic M90I allele (*13*, *33*). Therefore, RIT1-mediated activation of RAF is limited by factors unrelated to their binding affinity. One possibility is that RIT1 does not properly engage the cysteine-rich domain (CRD) of RAF, an essential step for productive RAF activation (*25*). A sequence of alignment of RIT1 with the classical RAS paralogs showed minimal conservation to the RAS residues critical for CRD binding, most of which are located at the interswitch region of RAS (fig. S6A). In addition, we modeled the interaction of RIT1 with the CRD domain of RAF1 using the solved KRAS:RAF1(RBD-CRD) crystal structure (fig. S6B) (*25*). Our model structure predicted that residues in the interswitch region of RIT1 (Arg^61^, Arg^63^, and Asp^66^) would cause important steric clashes and unfavorable interactions at the RIT1:RAF1(CRD) interface precluding RAF activation.

**Fig. 7.**
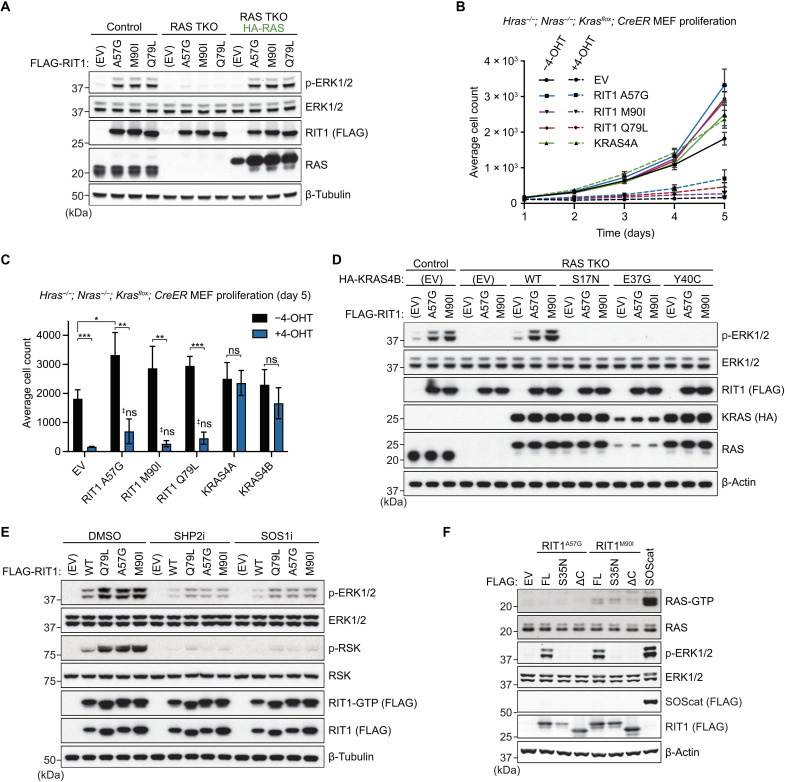
Pathogenic RIT1 relies on RAS to potentiate MAPK signaling. (**A**) Immunoblot analysis of indicated proteins from Rasless (HRAS/NRAS/KRAS TKO) or control HEK293 cells transiently transfected with indicated FLAG-tagged RIT1 constructs or an EV control and serum-starved for 16 hours. Rasless cells were rescued with ectopic expression of HA-tagged HRAS, NRAS, KRAS4A, and KRAS4B (1:1:1:1 DNA ratio). One of two independent experiments is shown. (**B**) Proliferation curves of control (−4-OHT) and Rasless (+4-OHT) MEFs stably expressing indicated constructs. Data points indicate the means ± SEM, *n* = 3. (**C**) Relative cell growth of control (−4-OHT) and Rasless (+4-OHT) MEFs stably expressing indicated constructs at day 5 of growth assay as in (B). Data points indicate means ± SD (*n* = 3); two-sided Student’s *t* test, *P ≤ 0.05, **P ≤ 0.01, and ***P ≤ 0.001; ‡ns, not significant (*P* > 0.05, versus EV + 4-OHT). (**D**) Immunoblot analysis of indicated proteins from TKO or control HEK293 cells transiently transfected with indicated FLAG-tagged RIT1 constructs or EV control and serum-starved for 16 hours. TKO cells were rescued with ectopic expression of indicated HA-tagged KRAS4B constructs or an EV control. One of two independent experiments is shown. (**E**) Immunoblot analysis of indicated proteins from HEK293T cells transiently transfected with indicated FLAG-tagged RIT1 constructs or an EV control and treated with 10 μM RMC-4550 (SHP2i), BI-3406 (SOS1i), or DMSO for 1 hour. GTP-bound RIT1 was precipitated with immobilized RGL3-RBD. One of two independent experiments is shown. (**F**) Immunoblot analysis of indicated proteins from HEK293 cells transiently transfected with indicated FLAG-tagged constructs and serum-starved for 16 hours. GTP-bound RAS was precipitated with immobilized RAF1-RBD. SOScat (SOS1 amino acids 564 to 1049). One of two independent experiments is shown.

Given that other nonclassical RAS GTPases, including Muscle RAS (MRAS) (*34*, *35*), promote RAF activation in a RAS-dependent manner, we hypothesized that pathogenic RIT1 may also rely on classical RAS proteins to activate RAF. Therefore, to evaluate RAS dependency, we knocked down HRAS, NRAS, and KRAS in WT or RIT1^M90I^ expressing primary mouse embryonic fibroblasts (MEFs). As expected, RAS knockdown attenuated growth factor–induced MAPK activation (fig. S7A). Moreover, RIT1^M90I^ enhanced the ERK signaling response compared to WT, and its effect was attenuated by RAS knockdown. However, despite our best efforts to deplete RAS using RNAi, this approach’s inefficiency did not allow for a proper evaluation of RAS dependency. Therefore, we generated human embryonic kidney (HEK) 293 cells devoid of classical RAS proteins via CRISPR-Cas9–mediated triple knockout (TKO) of *HRAS*, *NRAS*, and *KRAS*, which rendered them insensitive to receptor tyrosine kinase (RTK)–mediated MAPK pathway activation (fig. S7, B and C). This “Rasless” HEK293 TKO system was then used to evaluate the role of RAS proteins in mutant RIT1-MAPK signaling. Ectopic expression of two pathogenic RIT1 alleles (A57G and M90I) and RIT1^Q79L^ activated MAPK signaling in control cells but not in TKO cells (Fig. 7A). Ectopic rescue of RAS expression in TKO cells reinstated RIT1-mediated MAPK signaling (Fig. 7A), suggesting that in this cell system, RIT1 relies on RAS proteins to activate the MAPK pathway and can do so with any classical RAS protein (fig. S7D). Furthermore, the addition of the dominant-negative S35N mutation or a C-terminal deletion mutant confirmed that MAPK activation in control cells was dependent on nucleotide loading and proper localization of RIT1 to the PM, respectively (fig. S7E). To confirm that the absence of MAPK activation by mutant RIT1 in the absence of RAS was not due to the loss of ERK-mediated feedback, ERK activity was pharmacologically inhibited in parental HEK293T cells but did not abate RIT1-mediated MEK activation (fig. S7F). Rather, relief of negative feedback potentiated MEK activation downstream of mutant RIT1.

To corroborate our findings in an independent model, we used MEFs that can be rendered Rasless upon treatment with 4-hydroxytamoxifen (4-OHT) (*35*). Upon genetic deletion of all three endogenous RAS genes, stably expressed pathogenic RIT1 variants or RIT1^Q79L^ failed to rescue MAPK pathway activation in response to fetal bovine serum (FBS) stimulation unlike ectopically expressed WT KRAS4A or KRAS4B (fig. S7G). Because Rasless MEF proliferation is MAPK dependent (*35*), we assessed cell growth as an additional readout of MAPK activity (Fig. 7B). Ectopic expression of mutant RIT1 failed to rescue Rasless cell growth; however, we note that a trend toward a partial rescue, most noticeable with RIT1^A57G^, was observed (Fig. 7C). Moreover, mutant RIT1 expression enhanced the proliferation rate of control MEFs, consistent with RIT1-mediated MAPK pathway activation observed here and in other cell models with endogenous Ras expression (*6*, *11*, *13*, *36*). These data suggest that although mutant RIT1 is capable of direct RAF binding, its ability to activate the MAPK pathway requires classical RAS proteins.

### RIT1 activates MAPK signaling by promoting RAS-RAF binding

To further interrogate the role of RAS in the activation of MAPK signaling by RIT1, we rescued RAS expression in HEK293 TKO cells with WT KRAS4B, nucleotide binding–deficient KRAS4B^S17N^, or two RAF binding–deficient mutants (E37G and Y40C) (*37*, *38*), which, unlike WT KRAS4B, failed to promote RIT1-mediated MAPK activation (Fig. 7D). Furthermore, pharmacological inhibition of upstream RAS regulators SH2 containing protein tyrosine phosphatase-2 (SHP2) and SOS1, attenuated RIT1-mediated MAPK activation, despite having no effect on RIT1-GTP levels (Fig. 7E). Together, these data suggest that RAS nucleotide cycling and RAS:RAF binding are necessary for MAPK pathway activation by mutant RIT1. To rule out indirect pathway activation upstream of RAS (e.g., through the regulation of positive RAS regulators, such as SHP2 or SOS1/SOS2), we measured RAS-GTP levels from cells expressing pathogenic RIT1 and observed no increase in GTP-loaded RAS that correlated with MAPK pathway activation (Fig. 7F).

**Fig. 8.**
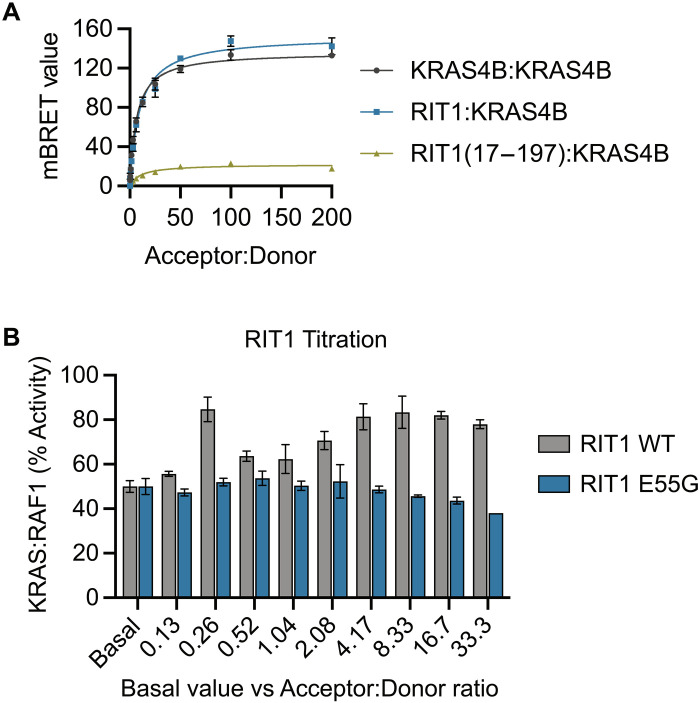
Aberrant RIT1 expression promotes RAS:RAF binding. (**A**) BRET binding curves show the association of HaloTag-KRAS4B or RIT1 (acceptor) and KRAS4B-nanoLuc (donor) proteins. Representative BRET curves from two independent experiments are shown. Data points indicate the means ± SD of technical triplicates. (**B**) BRET_50_ values determined by KRAS4B:RAF1 association in a nanoBRET assay with titration of RIT1 WT or E55G, normalized to basal (no RIT1 cotransfection) level set to 50% (% activity). Data points indicate the means ± SD (*n* = 3).

Given the requirement of RAS:RAF binding for MAPK activation downstream of RIT1 and no modulation of RAS GTP loading by mutant RIT1 expression, we posited that RIT1 molecules may promote RAS-RAF association by recruiting and increasing the local concentration of RAF at the PM near RAS molecules. To test this hypothesis, we used BRET to assess whether RIT1 proteins colocalize with RAS at the PM. The binding curves produced by KRAS:RIT1 and KRAS:KRAS proximity exhibited similar BRET_max_ and BRET_50_ values (Fig. 8A). In contrast, the proximity of KRAS with a cytoplasmic localized G domain of RIT1 (17 to 197) produced a drastically weaker binding response. These data suggest that RIT1 resides in close proximity to KRAS at the PM and is mediated by the RIT1 HVR. We next examined whether high RIT1 expression levels, akin to those resulting from pathogenic RIT1 mutations, modulate KRAS:RAF1 binding. Increasing levels of RIT1, but not RIT1^E55G^ (a RAF-binding deficient mutant), enhanced the apparent KRAS:RAF1 binding affinity in a BRET assay (Fig. 8B), indicating that RIT1 promotes RAS:RAF interaction in a manner dependent on RIT1:RAF1 binding. Together, these data indicate that RIT1 does not directly activate RAF but rather promotes its activation by facilitating RAS association. It further indicates that pathogenic RIT1-MAPK signaling may be susceptible to therapies that target either RAS exchange or canonical RAS-MAPK pathway components.

### MEK inhibition attenuates pathological RIT1 MAPK activation and hypertrophy in cardiac tissues

**Fig. 9.**
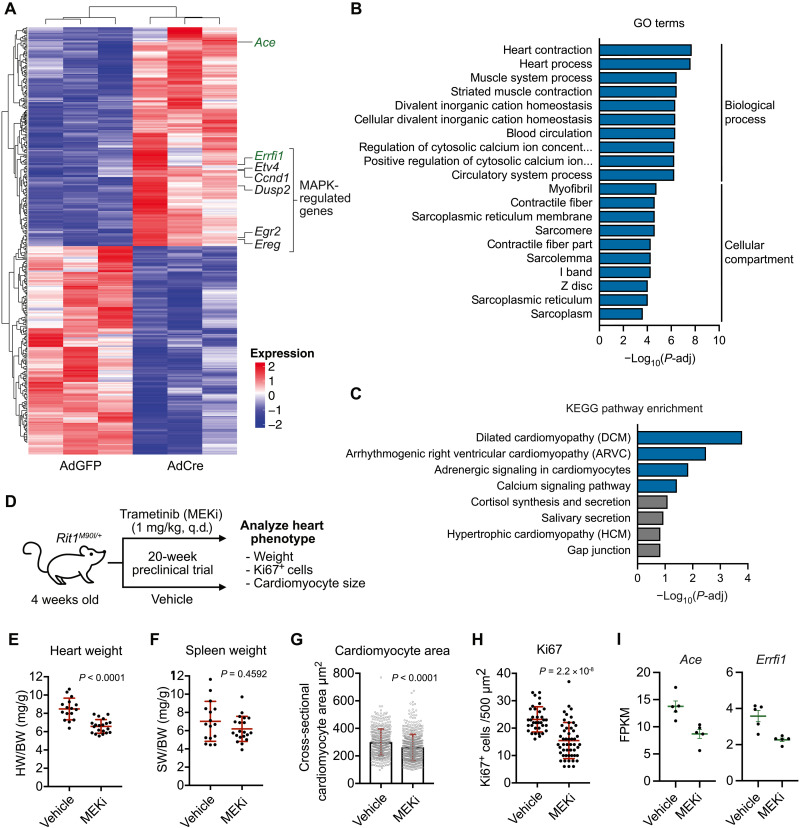
MAPK inhibition alleviates RIT1-dependent cardiac hypertrophy. (**A**) Heatmap of top differentially expressed genes in primary cardiomyocytes from *Rit1^LoxP-M90I^* neonates treated with adenovirus encoding Cre recombinase (AdCre) or GFP (AdGFP). (**B** and **C**) GO and KEGG enrichment analysis of differential gene expression elicited by RIT1^M90I^ expression in primary cardiomyocytes (AdCre versus AdGFP). (**D**) Schema of 20-week trametinib (MEKi) preclinical trial with *Rit1^M90I/+^* mice. q.d., once a day. (**E** and **F**) Comparison of normalized heart (E) or spleen (F) weight between MEKi (*n* = 16) and vehicle control (*n* = 20) group. Statistical significance was assessed by a two-tailed Mann-Whitney test. Error bars indicate means ± SD. (**G** and **H**) Quantification of myocyte area (G) and Ki67^+^ cells (H) from heart cross sections by immunofluorescence and immunohistochemistry, respectively. Statistical significance was assessed by a two-tailed Mann-Whitney test. Error bars indicate means ± SD. (**I**) Normalized mRNA transcript levels [Fragments Per Kilobase Million (FPKM)] of indicated genes in hearts (*n* = 5) isolated from vehicle control or MEKi-treated *Rit1^M90I/+^* mice at 20-week end point. Error bars indicate means ± SEM.

Mutations in pathway components upstream (SHP2 and SOS1) and downstream (RAF1 and SHOC2) of RAS that promote MAPK activation define the NS pathogenic landscape (*12*). The development of NS-associated congenital heart defects, a primary cause of morbidity and mortality, varies depending on the genetic driver. This is particularly notable in certain NS genotypes that are more likely associated with HCM, such as RAF1 and RIT1 NS (*9*, *12*). The high incidence of HCM (50 to 70%) and related heart defects (pulmonary stenosis and atrial septal defects) (*9*) in patients with RIT1 NS prompted us to establish an in vitro model system to investigate the impact of mutant RIT1 expression in cardiac cells. To this end, we isolated neonatal cardiomyocytes from mice harboring an engineered *Rit1* locus with Cre recombinase–inducible expression of the pathogenic variant *Rit1^M90I^* (*13*). Upon isolation, cardiomyocytes were treated with adenoviruses encoding for Cre recombinase to induce expression of the *Rit1^M90I^* variant. RNA sequencing (RNA-seq) analysis on day 6 after adenoviral delivery revealed that RIT1^M90I^ expression had elicited broad alterations in the transcriptomic landscape of cardiac myocytes (Fig. 9A). These changes included the up-regulation of several well-established MAPK target genes (*Ccnd1*, *Etv4*, *Egr2*, *Dusp2*, and *Ereg*), confirming that pathogenic RIT1 regulates MAPK signaling in this cell type (fig. S8A) (*39*). In addition, Gene Ontology (GO) and Kyoto Encyclopedia of Genes and Genomes (KEGG) analyses revealed an enrichment of genes critical for proper cardiac function (Fig. 9, B and C) and whose dysregulation may contribute to the cardiomyopathy-like phenotype exhibited by RIT1 NS murine models (*13*, *14*). Further, these data suggest that up-regulation of MAPK signaling by mutant RIT1 may drive the dysregulation of cardiomyopathy-associated genes, such as *Mybpc3* (myosin-binding protein C), a causal gene representing approximately 20% of patients with HCM (*40*), and *Actc1* (cardiac α-actin), among others (fig. S8B) (*40*, *41*).

Given the data above, we leveraged our RIT1 NS mouse model to evaluate whether pharmacological inhibition of the MAPK pathway may ameliorate RIT1^M90I^-driven cardiac tissue hypertrophy (*13*). To this end, we treated a cohort of 4-week-old mice harboring a germline *Rit1^M90I^* variant with the allosteric MEK1/MEK2 inhibitor (MEKi) trametinib or vehicle control (Fig. 9D). After 20 weeks of daily treatment, we observed a substantial decrease in heart weight of MEKi-treated mice (Fig. 9E) but no difference in spleen weight (Fig. 9F), suggesting that MEK inhibition may reduce aberrant cardiac tissue growth associated with mutant RIT1 expression. When the size (cross-sectional area) and proliferative state (Ki67 staining) of myocytes from MEKi and control hearts were compared, MEKi-treated hearts exhibited a marked reduction in both parameters, indicating reduced cell growth (Fig. 9, G and H). Transcriptomic profiling by RNA-seq confirmed that systemic MEKi treatment effectively inhibited MAPK signaling and suggests that the observed reduction in cardiac cell growth was a direct consequence of MEK inhibition within *Rit1^M90I/+^* hearts (fig. S8C). Together, these data suggest that pharmacological inhibition of aberrant RIT1-mediated MAPK signaling may represent a viable therapeutic strategy to ameliorate the cardiac defects presented by patients with RIT1 NS. Further analysis of our differential gene expression datasets identified two genes, *Ace* (angiotensin-converting enzyme 1) and *Errfi1* (ERBB receptor feedback inhibitor 1), which were transcriptionally up-regulated upon RIT1^M90I^ expression in primary cardiomyocytes (see Fig. 6A), and down-regulated in *Rit1^M90I/+^* hearts following MEKi treatment (Fig. 9I). These patterns of expression mark *Ace* and *Errfi1* as potential biomarkers for RIT1 NS individuals with associated HCM.

## DISCUSSION

Activation of the MAPK pathway occurs at the inner leaflet of the PM wherein lipid-anchored RAS GTPases recruit RAF kinases and facilitate a multistep activation process, resulting in active RAF dimers (*30*). Because the discovery of RIT1 and its paralog RIT2, the absence of HVR prenylation motifs prompted early speculation into their unique HVR-dependent PM association (*42*). Here, we show that RIT1, similar to the classical RAS GTPases, requires membrane binding for pathological MAPK activation. An extended polybasic HVR, containing three PBRs, mediates electrostatic interaction with negatively charged phospholipids, a property akin to the polybasic KRAS4B HVR; however, the absence of a RIT1 HVR lipid anchor may allow for transient and dynamic association with the PM. We have shown that RIT1 diffuses between PM and cytoplasm during mitosis to interact with spindle assembly checkpoint proteins mitotic arrest deficient 2 (MAD2) and p31^comet^, a process that is regulated by Cyclin-dependent kinase 1 (CDK1)–mediated HVR phosphorylation (*31*). Furthermore, appending a C-terminal prenylation motif prevents dissociation from the PM and blocks RIT1 mitotic regulation. We and others (*23*) have identified noncharged residues (Trp^204^, Leu^207^, and Phe^211^) interspersed between the PBRs critical for membrane association. Molecular dynamic simulations have revealed that the hydrophobic side chains of these residues bury deeply into the lipid bilayer (*22*); however, further investigation is needed to determine whether these residues help coordinate the association of PBRs with phospholipid head groups. We speculate that the uniquely electrostatic association with the PM may enable RIT1 to sense the composition of inner leaflet lipids.

To best interrogate the contribution of membrane association with RAF binding, we developed a BRET assay to quantitate RIT1-RAF association in the context of a native PM environment. We found that perturbations of the RIT1 HVR that abrogated membrane association strongly correlated with decreased RAF binding. Although anticipated, these data exemplify the critical nature of the RIT1 HVR in mediating RIT1’s diverse functions. In contrast with previous reports demonstrating that RIT1 associates preferentially with BRAF in neuronal cells (*17*), we found that RIT1 binds most strongly with RAF1 in vitro and in intact cells, suggesting that despite its increased affinity to RAF1, selectivity toward RAF paralogs in cells may be context dependent. Preferential RAF1 binding is a property also shared by the classical RAS family members in cells (*24*).

Accumulation of RIT1 through the loss of LZTR1-mediated proteasomal degradation increases MAPK signaling, a defining feature of RASopathies (*13*, *15*, *43*). However, as with other NS germline mutants, pathologic RIT1 signaling is mild and thus compatible with embryonic development (*1*). Despite reporting by independent groups of a direct, albeit weak, interaction between RIT1 and RAF kinases (*13*, *17*, *44*), evidence of RAF activation was limited. The data presented here identify RAF kinases, namely, RAF1, as the primary effectors through which mutant RIT1 proteins activate MAPK signaling. Nonetheless, it remains possible that RIT1 activates ERK1/ERK2 through alternate MAP3 kinases (*36*) and merits further investigation to determine whether different cell contexts determine effector selectivity.

**Fig. 10.**
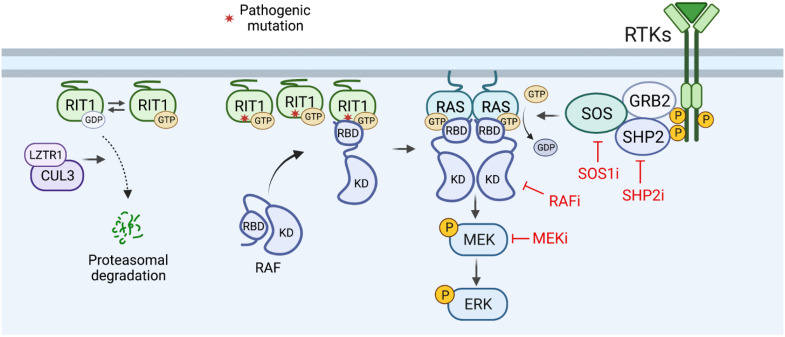
Model of MAPK pathway activation by mutant RIT1. Cancer and NS-associated pathogenic RIT1 variants promote MAPK pathway activation in a classical RAS-dependent manner. Mutant RIT1 proteins evade LZTR1-mediated proteasomal degradation and accumulate at the PM residing in close proximity to RAS. Pathogenic RIT1 accumulation drives RAF recruitment to the PM via the RAF-RBD but inefficient CRD engagement limits RAF activation. However, the increased local concentration of RAF promotes its activation by RAS molecules in response to upstream RTK signaling. Inhibition of various RTK-RAS-MAPK signaling components (e.g., SHP2, SOS1, RAF, and MEK) abated aberrant MAPK activation by mutant RIT1. KD, kinase domain.

Approximately one-fifth of individuals with RIT1 NS harbor an A57G allele, making it the most common *RIT1* variant in this condition (*8*). RIT1^A57G^ is of particular interest, biochemically, due to its neomorphic enhanced binding to RAF kinases, shown above, while exhibiting comparable rates of intrinsic GTP hydrolysis, nucleotide exchange, and cellular fraction bound to GTP as other gain-of-function alleles (*13*, *44*). Intriguingly, RIT1^A57G^ activates MAPK signaling to a similar extent as other pathogenic variants despite an increased affinity to RAF (*13*, *33*). Thus, RIT1 activation of RAF is not solely correlated to the strength of their interaction, suggesting that this is not the limiting factor associated with the modest degree of MAPK pathway activation exhibited by pathogenic *RIT1* variants. The CRD of RAF1 makes critical contacts with the interswitch region of KRAS that are essential for RAF activation (*25*). We speculate that the interswitch region of RIT1, which shares low homology with the RAS interswitch region, likely fails to engage productively with the CRD of RAF limiting RAF activation upon RIT1 binding. We have found that activation of MAPK requires classical RAS proteins, consistent with the fact that deletion of these proteins in mouse cells results in a complete growth arrest (*35*). Furthermore, mutant RIT1 expression did not influence the proportion of GTP-loaded RAS, suggesting that RIT1 promotes MAPK pathway activation downstream of RAS while remaining dependent on RAS activation, cycling, and RAS:RAF binding. We posit that despite the low RIT1:RAF binding affinity, the overabundance of mutant RIT1 protein resulting from the pathogenic evasion of proteasomal degradation may facilitate RAF recruitment to the PM by mass action (Fig. 10). Because RIT1 is in proximity with KRAS, potentially as part of KRAS nanoclusters via their shared affinity for anionic lipids (*45*), recruitment of RAF to the PM by mutant RIT1 may increase the local concentration of RAF molecules “primed” for RAS activation in response to upstream RTK signaling and RAS exchange. Although further investigation is needed to shed light on the exact mechanism, our findings indicate that RIT1-driven disease may be treated with RAS-MAPK pathway inhibitors, including those that block RAS activation (SHP2i or SOS1i) or with inhibitors currently in development that target RAS-GTP.

Compared to patients with other genetic variants, individuals with RIT1 NS exhibit an elevated frequency of HCM (*9*, *12*). In a prior study, we described a RIT1^M90I^ mouse model that recapitulated clinical manifestations of NS disease, including cardiac hypertrophy (*13*). Here, we demonstrate that treatment of RIT1^M90I^ mice with the U.S. Food and Drug Administration–approved MEK1/MEK2 inhibitor trametinib (GSK1120212) ameliorated cardiac tissue overgrowth, suggesting that targeting the MAPK pathway may be an effective therapeutic strategy in NS patients with mutant RIT1. Off-label trametinib treatment was recently shown to reverse myocardial hypertrophy in three children with RIT1 NS (*46*, *47*). However, trametinib has been reported to induce substantial levels of toxicity in other disease contexts (*48*, *49*), highlighting the need for further preclinical work to address optimal dosing and treatment windows, response to different MEK inhibitors, and efficacy of upstream RAS inhibition to alleviate the cardiac and extracardiac RIT1 phenotype. Together, these findings aid our mechanistic understanding of RIT1 disease and support the evaluation of broader therapeutic strategies.

Last, we must consider the implications of a RAS-dependent RIT1 MAPK activation in the context of RIT1-driven tumors. Cells expressing RIT1^M90I^, but not those expressing KRAS^G12V^, depend on RTK and adaptor proteins upstream of RAS, including epidermal growth factor receptor (EGFR), Growth Factor Receptor Bound Protein 2 (GRB2), SHP2, and SOS1 for growth (*50*). Conversely, loss of NF1 and Sprouty Related EVH1 Domain Containing 1 (SPRED1_, two negative regulators of RAS activity, promote mutant RIT1 cell growth, consistent with our model in which RIT1 relies on active RAS to promote MAPK signaling. In the same system, Vichas *et al.* (*50*) show that loss of LZTR1 similarly promotes the growth of cells expressing ectopic RIT1^M90I^, suggesting that stabilization of endogenous RIT1 may provide an additional growth advantage (*13*). In certain cell contexts, LZTR1 promotes the degradation of other RAS family GTPases, most notably MRAS (*13*, *51*), and its loss may enhance MRAS-mediated RAF activation in synergy with mutant RIT1 expression (*52*). In support of this hypothesis, mutant RIT1 cells exhibited a strong growth dependency on SHOC2, a scaffolding protein that associates with MRAS to promote RAS-dependent activation of RAF (*53*). In addition, RIT1 moonlights as a mitotic checkpoint regulator (*31*, *50*), among other functions (*8*, *33*), imparting mutant RIT1 cancer cells with unique therapeutic vulnerabilities (*50*). While our work sheds some light on RIT1’s dependency on RTK-MAPK components, further studies are needed to define the contribution of RIT1’s various functions to its oncogenic potential.

## MATERIALS AND METHODS

### Protein production

#### Cloning

DNA constructs for the expression of KRAS4B (1 to 169) (Addgene, #159539) and RAF1 (52 to 131) were previously described (*54*). Gateway entry clones for BRAF (151 to 277), RIT1 (17 to 197), RIT1 A57G (17 to 197), and RIT1 (17 to 219) were generated by standard cloning methods and incorporate an upstream tobacco etch virus (TEV) protease cleavage site (ENLYFQG) followed by the appropriate coding sequences. Sequence-validated entry clones were subcloned into pDest-566, a gateway destination vector containing a His6 and maltose-binding protein (MBP) tag to produce the final *Escherichia coli* expression clones (*55*). All expression constructs were in the form containing an N-terminal His6 and MBP tag (Addgene, #11517).

#### Protein expression

RAF1-RBD (52 to 131) was expressed using the autoinduction media protocol, and BRAF (151 to 277) and RIT1 proteins were expressed using the Dynamite media protocol (*55*). For ^15^N isotopic incorporation into RAF1 (52 to 131), KRAS4B (1 to 169), RIT1 (17 to 197), RIT1 (17 to 219), and RIT1 (17 to 197) A57G, seed cultures were inoculated from glycerol stocks of the transformed strains into 300 ml of Studier’s MDAG135 medium (*56*) (25 mM Na_2_HPO_4_, 25 mM KH_2_PO_4_, 50 mM NH_4_Cl, 5 mM Na_2_SO_4_, 2 mM MgSO_4_, 50 μM FeCl_3_, 20 μM CaCl_2_, 10 μM MnCl_2_-4H_2_O, 10 μM ZnSO_4_-7H_2_O, 2 μM CoCl_2_-6H_2_O, 2 μM CuCl_2_-2H_2_O, 2 μM NiCl_2_-6H_2_O, 2 μM Na_2_MoO_4_-2H_2_O, 2 μM Na_2_SeO_3_-5H_2_O, and 2 μM H_3_BO_3_), 19.4 mM glucose, 7.5 mM aspartate, and 200 μg/ml each of 18 amino acids (E, D, K, R, H, A, P, G, T, S, Q, N, V, L, I, F, W, and M) in a 2-liter baffled shake flask for 16 hours at 37°C until late-log phase growth. In the interim, 15 liters of T-20052 (*57*) medium was prepared in a 20-liter BioFlow IV bioreactor (Eppendorf/NBS). The seed culture was collected and centrifuged at 3000*g* for 10 min at 25°C. The pellet was resuspended with 100 ml of the sterilized T-20052 medium from the bioreactor and then returned to the bioreactor as inoculum. The culture was grown at 37°C with an airflow of 15.0 liters per minute (LPM) and agitation of 350 rpm. Approximately 5 hours (mid-log phase) after inoculation, the culture was shifted to 20°C overnight. Cells were collected by centrifugation (5000*g* for 10 min at 4°C), and the cell pellet was stored at −80°C.

#### Protein purification

Proteins were essentially purified as previously described for proteins in the His6-MBP-TEV-POI (protein of interest) expression format (*58*). Note that 5 mM MgCl_2_ was used in buffers used to purify KRAS4B (1 to 169). Essentially, the His6-MBP-POI was purified by immobilized metal-ion affinity chromatography (IMAC) from the lysate, the His6-MBP tag was removed by His6-TEV protease digestion, the POI was isolated from the TEV digest by another round of IMAC (POI in flow-through, wash, or low imidazole elutions), the pooled protein was buffer-exchanged via preparative size exclusion chromatography, concentrated and frozen in liquid nitrogen in aliquots, and stored at −80°C.

Purifications of RIT1 required some alterations to this basic protocol. Specifically, protein concentration was kept below 4 mg/ml throughout, and all ^15^N preparations of RIT1 (17 to 197) and preparations of RIT1 (17 to 219) were with 300 mM NaCl and 10% (w/v) glycerol in all buffers. Final buffers were 20 mM Hepes (pH 7.3), 150 mM NaCl, 1 mM tris(2-carboxyethyl)phosphine (TCEP) for BRAF (151 to 277), 10 mM tris-HCl (pH 7.5), 50 mM NaCl, 2 mM MgCl_2_ for RAF1 (52 to 131), 20 mM Hepes (pH 7.3), 150 mM NaCl, 2 mM MgCl_2_, 1 mM TCEP for RIT1 (17 to 197), and KRAS4B (1 to 169) with 5 mM MgCl_2_ added for KRAS concentrations greater than 10 mg/ml and 20 mM Hepes (pH 7.3), 300 mM NaCl, 2 mM MgCl_2_, 1 mM TCEP, 10% glycerol for RIT1 (17 to 219), and ^15^N preparations of RIT1 (17 to 197).

### Liposome SPR

Liposomes (2.5 mM) were prepared from various amounts of 1-palmitoyl-2-oleoyl-glycero-3-phosphocholine (POPC) and POPS. Lipid mixtures were lyophilized at −80°C for approximately 3 hours. Lipids were reconstituted in 1 ml of 20 mM Hepes (pH 7.4) and 150 mM NaCl buffer and sonicated at 37°C for 5 min. Mixtures underwent five freeze/thaw cycles, followed by another brief sonication until clear.

SPR-binding experiments were run on a Biacore T200. Liposomes composed of POPC or POPS were captured on flow cells 2 to 4 of an L1 Chip, and flow cell 1 was unmodified and served as a reference. RIT1 was diluted in SPR running buffer [20 mM Hepes (pH 7.4) and 150 mM NaCl], concentration range, 5 to 0.156 μM (1:2-fold dilutions), and injected on the chip surface at 30 μl/min. Sensorgrams were normalized by the capture level of liposomes. Partition coefficients were calculated as described previously (*59*).

### GNP exchange

RIT1 (2 mg) was diluted in alkaline phosphatase buffer [40 mM tris (pH 7.4), 200 mM ammonium sulfate, 1 mM ZnCl_2_, and 10% glycerol]. GppNHp {Guanosine-5'-[(β,γ)-imido]triphosphate} (10 mM) and 5 U of alkaline phosphatase beads were added to RIT1, and the mixture was incubated for 1 hour at 4°C with constant rotation. Beads were pelleted out, and 30 mM MgCl_2_ was added to the mixture, followed by another brief incubation at 4°C. Protein was desalted on a HiPrep 26/10 desalting column into buffer [20 mM Hepes (pH 7.4), 150 mM NaCl, 5 mM MgCl_2_, and 1 mM TCEP]. The efficiency of exchange was measured by high-performance liquid chromatography analysis, as previously published (*57*).

### Isothermal titration calorimetry

ITC experiments were run on a MicroCal PEAQ-ITC instrument. The proteins were diluted to 100 μM (RIT1) and 300 μM (RAF) in 20 mM Hepes, 150 mM NaCl (pH 7.4), 5 mM MgCl_2_, and 1 mM TCEP buffer. Approximately 200 μl of RIT1 was loaded into the cell of the instrument, and 80 μl of RAF was loaded into the syringe. RAF1 (4.4 μl) was titrated onto RIT1 every 2 min, for a total of 19 injections. After all injections were complete, the data were analyzed using the MicroCal PEAQ-ITC analysis software to calculate the *K*_D_.

### Nuclear magnetic resonance

RAF1-RBD (52 to 131), RIT1(17 to 197) WT/A57G, and WT KRAS4B (2 to 169) as well as their complexes were characterized by solution NMR spectroscopy (see fig. S4). For all these proteins, published backbone assignments were used initially with in-home NMR assignments whenever required [BMRB IDs: RAF1 (17382), RIT1 (26787), and KRAS (28021)]. All ^15^N-labeled proteins used in this study had concentrations between 100 and 150 μM. Protein-protein complexes were preformed at a 1:3 ratio (threefold excess of unlabeled partner), and the saturating complex was confirmed by observing the signal of the key reporting residues. Two-dimensional (2D) ^1^H-^15^N heteronuclear single-quantum coherence (HSQC) spectra were recorded at 25°C on a Bruker 700-MHz spectrometer equipped with proton-cooled cryogenic ^1^H/^13^C/^15^N triple-resonance probes. The sample temperature was calibrated with a 100% methanol sample before the experiments. All ^1^H-^15^N HSQC spectra were collected with 1000 and 128 complex points in F2 (^1^H) and F1(^15^N), respectively, with 16 scans. All experiments used a ^1^H spectra width of 9090 Hz and a ^15^N spectral width of 1987 Hz with the proton and ^15^N carriers set to 4.7 and 120 parts per million. Data were processed by NMRPipe (*60*) and analyzed by NMRFAM-SPARKY (*61*), GraphPad Prism was used to plot CSP plots, and PyMOL was used to map CSP to x-ray structures. CSPs were calculated by using the equations Sqrt{[dH^^2^ + (dN/10)^^2^]/2}, where dH and dN are the proton and nitrogen chemical shift differences between the complexed and noncomplexed proteins.

### Cell lines and culture conditions

HeLa and HEK293T cells were obtained from the American Type Culture Collection. FlpIn HEK293 cells were obtained from Thermo Fisher Scientific. *Hras^−/−^; Nras^−/−^; Kras^flox^; CreER* MEFs were originally developed by M. Barbacid’s laboratory and were provided by N. Fer (Frederick National Laboratory). Cells were cultured in Dulbecco’s modified Eagle’s medium (DMEM) supplemented with 10% FBS. Cells were grown in a humidified incubator with 5% CO_2_ at 37°C. Validation procedures are as described by the manufacturer. Cell lines were regularly tested and verified to be mycoplasma negative using the MycoAlert PLUS Mycoplasma Detection Kit (Lonza). RMC-4550, SCH772984, BI-3406, and LY3009120 were purchased from Selleckchem.

For the generation of Rasless 293 cells, crispr RNA (crRNA_–targeting HRAS [Integrated DNA Technologies (IDT)] and ATTO 550–labeled Trans-activating CRISPR (tracr) RNA (tracrRNA) (IDT) were combined to a final concentration of 1 μM and annealed by cooling from 95°C to room temperature. crRNA:tracrRNA duplex (12 pmol) was combined with 12 pmol of recombinant HiFi Cas9 (IDT) and reverse-transfected into HEK293 Flp-In cells using Lipofectamine CRISPRMAX reagent (Thermo Fisher Scientific) following IDT protocols. Twenty-four hours after transfection, ATTO 550–positive cells were sorted using a Sony SH800 Cell Sorter and allowed recovery for 4 days. Following the same procedure, cells were then sequentially transfected with NRAS and KRAS crRNA:tracrRNA-Cas9 ribonucleoprotein complexes. For the final cell sorting step following KRAS crRNA:tracrRNA-Cas9 transfection, single ATTO 550–positive cells were sorted into 96-well plates. Clonal cell lines were then expanded and screened for HRAS, KRAS, and NRAS knockout by Western blotting using antibodies specific for the individual RAS paralogs and two pan-HRAS/KRAS/NRAS antibodies. Five confirmed TKO clones were then pooled to mitigate off-target guide RNA effects and clonal heterogeneity and validated functionally through the absence of MAPK signaling. To generate HEK293 Flp-In control single guide RNA (sgRNA) cells, the same procedures were performed in parallel targeting safe loci in *AAVS1*, chromosome 3 and chromosome 15 (*62*). Single-cell clones were expanded, and the normal activity and expression of RAS signaling pathway components were confirmed by immunoblotting. Twelve clones were then pooled to give a polyclonal control sgRNA cell line. See table S2 for sgRNA targeting sequences.

For the generation of Rasless MEFs with stable expression of ectopic RIT1 or RAS, lentivirus was produced by cotransfection of HEK293T cells with a lentiviral vector and the packaging plasmids psPAX2 (Addgene, plasmid #12260) and pMD.2G (Addgene, plasmid #12259) at a ratio of 1.25:1.0:0.25. The supernatant was collected 72 hours after transfection and filtered through a 0.45-μm filter. *Hras^−/−^; Nras^−/−^; Kras^flox^; CreER* MEFs were transduced with lentiviral-containing supernatant supplemented with polybrene (0.8 μg/ml; Sigma-Aldrich). Stably transduced cells were selected with puromycin (1.5 μg/ml; Sigma-Aldrich). To remove the floxed *Kras* allele, stable cells were treated with 1 μM 4-OHT (Sigma-Aldrich). Assays with Rasless MEFs were conducted 10 to 11 days after 4-OHT treatment, and loss of KRAS was verified by immunoblot.

### Mammalian expression constructs

All RIT1 complementary DNA (cDNA) mutants were generated using standard polymerase chain reaction–based site-directed mutagenesis in the pDONR223-RIT1 template and were previously described (*31*). KRAS4A and KRAS4B entry clones (Addgene, plasmids 83166 and 83129) and ARAF, BRAF, and RAF1 (plasmids 70293, 70299, and 70497) were a gift from D. Esposito (Frederick National Laboratory). The catalytic domain of SOS1, SOScat, (residues 564 to 1049) was previously described (*15*). For N-terminal glutathione *S*-transferase (GST)–tagged proteins, entry clones were gateway-cloned into pDEST27 destination vector (Invitrogen). For N-terminal mCherry-, EGFP-, or FLAG-tagged constructs, entry clones were cloned by multisite gateway cloning into pDEST302 or pDEST663 (a gift from D. Esposito, Frederick National Laboratory) and with expression controlled by an EF1a promoter. Hemagglutinin (HA)–tagged constructs were gateway cloned into a pcDNA-HA destination vector. EV plasmid controls were generated using a gateway recombination cassette containing a stop codon followed by an untranslated stuffer sequence.

### RNA interference

To knockdown RAS, individual pools of short interfering RNAs (siRNAs) against mouse *Hras*, *Nras*, and *Kras* (SMARTpool: ON-TARGETplus, Horizon) were pooled in equal amounts and transfected into cells using Lipofectamine RNAiMax Transfection Reagent (Life Technologies) according to the manufacturer’s instructions. Similarly, knockdown of RAF kinases was achieved using siRNA-targeting human *RAF1*, *ARAF*, and *BRAF* (SMARTpool: ON-TARGETplus, Horizon). Nontargeting control siRNA was purchased from Horizon (D-001810-10-05).

### Primary cardiomyocyte isolation

Preparation of mouse neonatal cardiac myocytes was conducted as previously described (*63*) with some modifications. Briefly, hearts were extracted from 1- to 3-day-old neonates, placed in ice-cold Hanks’ balanced salt solution (HBSS), cleared of blood clots and aortic tissue, then placed in fresh ice-cold HBSS, and cut into small pieces to be digested in prewarmed HBSS with collagenase type 2 (1 mg/ml; Life Technologies) at 37°C for 5 min with gentle agitation. Heart pieces were then allowed to sediment for 1 min, and the supernatant was removed and discarded. The following digestion process was repeated six times: heart pieces from before were resuspended in fresh HBSS with collagenase (1 mg/ml) and incubated at 37°C for 5 min with gentle agitation. Heart pieces were then allowed to sediment for 1 min, and the supernatant containing suspended cardiac myocytes was transferred to a new tube containing 1/10 volume of cardiomyocyte medium [DMEM supplemented with 10% FBS, 10% Nu serum (Corning), 1× penicillin-streptomycin (Gibco), 1× GlutaMAX (Gibco), 1× ITS liquid medium supplement (Sigma-Aldrich), and 10 mM Hepes (Gibco)] and centrifuged to pellet cells (500*g* for 5 min). Cell pellets were resuspended in 1 ml of cardiomyocyte medium and pooled after the sixth collection, passed through a cell strainer multiple times, and incubated at 37°C for 2 hours in a plastic 10-cm dish. Unadhered cells in cell suspension were then pelleted by centrifugation and resuspended in 1 ml of cardiomyocyte medium per heart and seeded on a laminin-coated 12-well plate at a density of ~1 heart per well. Cells were exchanged into fresh media daily and infected with adenoviral particles encoding GFP or GFP-Cre (2 × 10^8^ per well; ViraQuest) on day 3.

### Reverse transcription quantitative polymerase chain reaction

Total RNA from cardiomyocytes was obtained on day 6 after adenovirus infection using an RNeasy kit (QIAGEN) according to the manufacturer’s instructions. cDNA was obtained by reverse transcription of 1 μg of RNA using qScript XLT cDNA SuperMix (QuantaBio, 95161). Ten nanograms of cDNA was diluted in nuclease-free water and ran in technical triplicates using PowerUp SYBR Green Master Mix (Applied Biosystems) on a QuantStudio 5 (Thermo Fisher Scientific). *Tbp* (TATA-box binding protein) was used as an endogenous control. See table S3 for primer sequences.

### Live-cell imaging

HeLa cells were seeded onto 12-well #1.5 glass-bottom plates (Cellvis) and transiently transfected with FuGENE 6 (Promega), following the manufacturer’s instructions. Before imaging, cells were exchanged into imaging media: FluoroBrite DMEM (Thermo Fisher Scientific) supplemented with 10% FBS and 4 mM GlutaMAX (Thermo Fisher Scientific). Images were acquired as a series of 0.6-μm z-stacks with a Plan Apo 40×/0.95 Corr [Differential Interference Contrast (DIC) N2/40× I] 227.5 nm per pixel objective (Nikon) on a Nikon Ti-E inverted CSU-22 spinning disk confocal microscope equipped with an incubation chamber (Okolab), providing a humidified atmosphere at 37°C with 5% CO_2_. Images were processed using Fiji (*64*).

### NanoBRET RIT1/KRAS-RAF interaction assay

HEK293T cells were seeded in a 12-well plate at 1.25 × 105 cells per well density. After 24 hours, cells were transfected with mVenus- or HaloTag-fusion (acceptor) contructs and nanoLuc-tagged (donor) using FuGENE 6. The concentration of donor was kept constant, and the concentration of acceptor was diluted twofold (concentrations range, 0 to 1000 ng). An EV plasmid was transfected into cells to normalize the DNA amount in each well. Forty eight hours after transfection, cells were trypsinized and recovered in DMEM cell medium containing 10% FBS. Tubes were spun down at 1500 rpm for 3 min, and the pellet was resuspended in Dulbecco’s phosphate-buffered saline (PBS) + 0.5% FBS. A cell suspension (20,000 cells) was added in triplicate to both a white 384-well Optiplate (Perkin Elmer) (for BRET reading) and a black 384-well plate (for mVenus reading). Twenty PBS + 0.5% FBS were added to each well of cells in the black plate to bring the final volume to 40 μl. Twenty microliters of 30 mM nanoBRET nano-Glo substrate (Promega) was added to all required wells of the 384-well PE Optiplate. Plates were read on a PerkinElmer Envision plate reader. The white plate was monitored at 535 nm (BRET signal) and 470 nm (background nanoLuc). mVenus was monitored in the black plate at a 530-nm emission with an excitation at 500 nm. The BRET value at each point was measured by dividing the BRET signal by the background nanoLuc signal. Acceptor/donor ratios were normalized against control with equal amounts of acceptor and donor transfected. Data were analyzed using a nonlinear regression fit with GraphPad Prism software to obtain the BRET_50_ values.

### Pulldowns and immunoblotting

For GST pulldown of proteins from cell lysates, 3 × 10^6^ HEK293T cells were transfected with 4 μg of total DNA of indicated plasmids. Twenty-four hours after transfection, cells were rinsed with ice-cold PBS and lysed with 1 ml of lysis buffer [50 mM tris-HCl (pH 7.5), 150 mM NaCl, 1% IGEPAL CA-630, and 10% glycerol] supplemented with protease and phosphatase inhibitor cocktails (Sigma-Aldrich). Lysates were cleared by centrifugation and incubated with 20 μl of Glutathione Sepharose 4B beads for 4 hours at 4°C with end-over-end rotation. Beads were rinsed three times with lysis buffer and resuspended in lithium dodecyl sulfate (LDS) sample buffer.

Whole-cell lysates for immunoblot analysis were prepared using radioimmunoprecipitation assay buffer [50 mM tris-HCl (pH 8.0), 150 mM NaCl, 0.5% sodium deoxycholate, 0.1% SDS, and 1% IGEPAL CA-630] supplemented with protease and phosphatase inhibitor cocktails (Sigma-Aldrich). Fifteen to thirty micrograms of total protein were loaded per well of precast NuPAGE gels (Life Technologies).

For immunoblot detection, samples were separated by SDS–polyacrylamide gel electrophoresis and transferred onto nitrocellulose membranes. Membranes were blocked using 5% skimmed milk in Tris-buffered saline with 0.1% Tween 20 (TBST) for 1 hour and incubated with appropriate primary antibodies overnight. Detection was performed using secondary antibodies conjugated to DyLight680 (1:10,000; 611-144-002) or DyLight800 (1:10,000; 610-145-002) (Rockland), visualized with a LI-COR Odyssey infrared scanner or using horseradish peroxidase–linked secondary antibodies, and developed with Amersham ECL (Cytiva Life Sciences) and x-ray films. Primary antibodies against p-ERK (1:1000 to 1:2000; 4370), ERK1/ERK2 (1:1000; 4696 and 4695), p-MEK (1:1000; 9154), MEK1/MEK2 (1:1000; 4694 and 8727), p-RSK (1:1000; 8753), RSK (1:1000; 9355), p-EGFR (1:1000; 3777), EGFR (1:1000; 4267), HA (1:1000; 3724), β-tubulin (1:1000; 2128), and FLAG (1:1000; 14793) were obtained from Cell Signaling Technology. Antibodies against GST (1:1000; sc-138) and HRAS (1:500; sc-520) were obtained from Santa Cruz Biotechnology. RIT1 (1:100; ab53720), NRAS (1:2000; ab167136), and pan-RAS (1:1000; ab108602) antibodies were from Abcam. KRAS (1:500; WH0003845M1), β-actin (1:10,000; A2228), ɑ-tubulin (1:5000; T6199), and FLAG (1:2000; F1804) antibodies were purchased from Sigma-Aldrich.

### Kinase assay

To measure RAF activity in vitro, FLAG-tagged RAF1 or BRAF was coexpressed with RIT1^A57G^, KRAS^Q61L^, or EV control in HEK293T cells. Twenty-four hours after transfection, cells were rinsed with ice-cold PBS and lysed with 1 ml of lysis buffer [50 mM tris-HCl (pH 7.5), 150 mM NaCl, 1% IGEPAL CA-630, and 10% glycerol] supplemented with protease and phosphatase inhibitor cocktails (Sigma-Aldrich). Lysates were cleared by centrifugation and incubated with 10 μl of anti-FLAG M2 agarose beads (EMD Millipore) for 4 hours at 4°C with end-over-end rotation. Beads were rinsed three times with lysis buffer and twice with Tris NaCl (TN) buffer [20 mM tris (pH 7.5), 150 mM NaCl, and 5% glycerol]. RAF protein was then eluted with 3xFLAG peptide (200 μg/ml; Sigma-Aldrich) in TN buffer, aliquoted, and snap-frozen in liquid nitrogen. Kinase reactions were performed as follows: 100 μl of reactions containing 2.5 nM FLAG-RAF1 or 1 nM FLAG-BRAF protein and 1 μM recombinant Avi-MEK1 (K97R), a gift from D. Esposito (Frederick National Laboratory); 20 mM tris (pH 7.5); 100 mM NaCl; 1 mM MgCl_2_; 1 mM dithiothreitol; and 0.5 mM adenosine 5′-triphosphate. A total of 20-μl fractions were removed at indicated time points, and the reaction was stopped by the addition of 2× LDS sample buffer.

### Mice

Conditional Rit1^M90I^ mice were previously described (*13*). To generate the experimental cohorts, conditional homozygous male Rit1^M90I^ mice were crossed to homozygous female cytomegalovirus-Cre deleter transgenic mice. Offspring were weaned at 3 weeks of age, and treatment was started at 4 weeks of age for a period of 20 weeks. Both male and female littermates were included in this study. Trametinib was purchased from Selleckchem and was diluted in 0.5% carboxymethylcellulose and 0.2% Tween 80 (Sigma-Aldrich). Upon completion of the 20 weeks, mice were given the last dose in the morning and euthanized 2 hours later. Body, heart, and spleen weight was recorded. Both the heart and spleen were fixed in phosphate-buffered formalin overnight. This study was performed in accordance with the guidelines in the *Guide for the Care and Use of Laboratory Animals* of the National Institutes of Health. All the animals were handled according to approved institutional animal care and use committee protocol no. AN165444 of the University of California, San Francisco (UCSF).

For the quantification of cardiomyocyte size, transverse cardiac tissue sections were stained with Texas Red–conjugated wheat germ agglutinin (1:200; W21405) to label the cell boundaries and counter-stained with 4′,6-diamidino-2-phenylindole. Five images of cardiac tissue adjacent to the left ventricle were captured at ×20 magnification for at least three mice per treatment group. The cross-sectional area of 30 cardiomyocytes per image was measured using Fiji.
