# Supplementary material for: RAS-dependent RAF-MAPK hyperactivation by pathogenic RIT1 is a therapeutic target in Noonan syndrome–associated cardiac hypertrophy
Source: Sci Adv. 2023 Jul 14;9(28):eadf4766. doi: 10.1126/sciadv.adf4766 (PMC10348673; doi:10.1126/sciadv.adf4766)
Supplement: Supplementary file 1 — Figs. S1 to S8 Tables S1 to S3 [file sciadv.adf4766_sm.pdf]

Science Advances
AAAS

Supplementary Materials for

RAS-dependent RAF-MAPK hyperactivation by pathogenic RIT1 is a therapeutic target in Noonan syndrome–associated cardiac hypertrophy

Antonio Cuevas-Navarro *et al.*

Corresponding author: Andrew G. Stephen, stephena@mail.nih.gov; Pau Castel, pau.castel@nyulangone.org

Sci. Adv. **9**, eadf4766 (2023)
DOI: 10.1126/sciadv.adf4766

This PDF file includes:

Figs. S1 to S8
Tables S1 to S3

Supplementary Figures

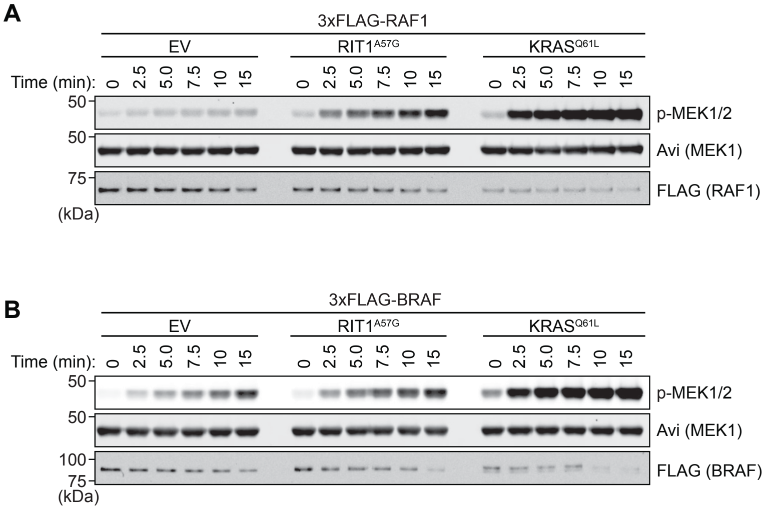

fig. S1. In vitro RAF kinase assay with RIT1^{A57G} co-expression

(**A, B**) Immunoblot analysis of kinase-dead MEK1 phosphorylation at indicated times by RAF1 (A) or BRAF (B) protein isolated from cells co-expressing RIT1^{A57G}, KRAS^{Q61L} or an empty vector (EV) control, in an in-vitro assay, see Methods.

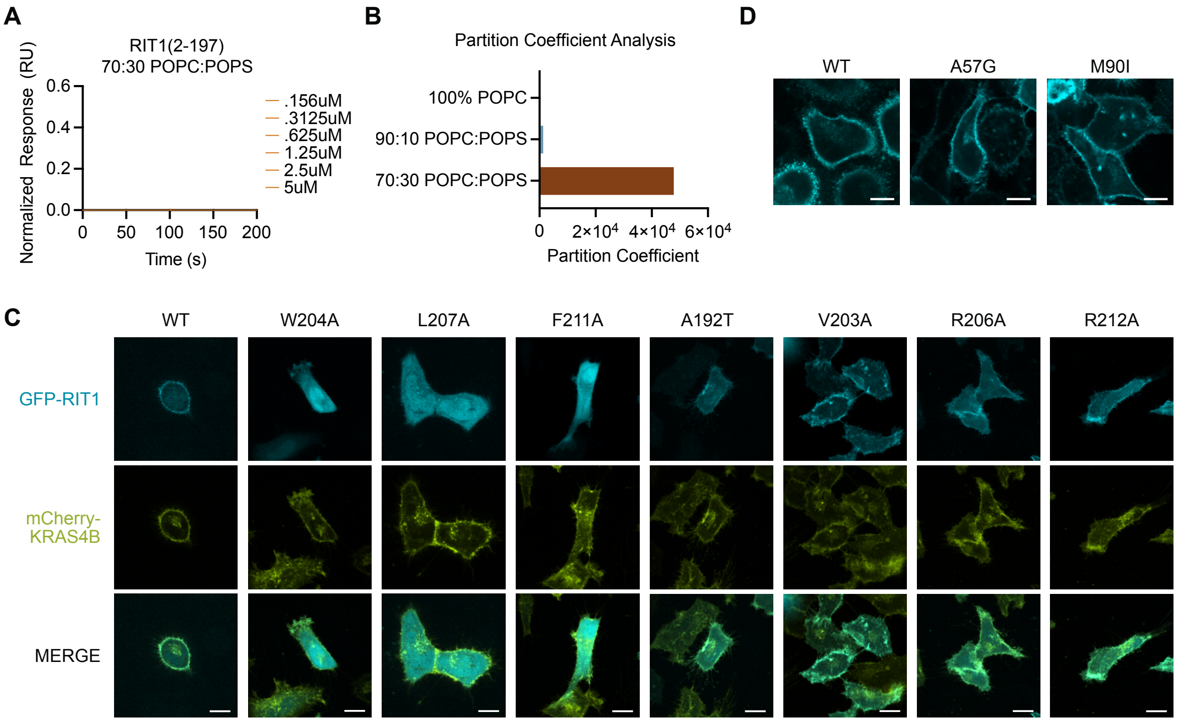

fig. S2. In vitro and in vivo analysis of RIT1-PM lipid associations

(A) SPR analysis shows no association between 30% POPS-containing liposomes and RIT1 protein (a.a. 2-197) with C-terminal deletion. (**B**) Partition coefficients derived from SPR affinity curves from Fig. 2B show the relative binding affinity of RIT1 to liposomes of indicated composition. (**C**) Live-cell confocal images of HeLa cells transiently transfected with indicated GFP-RIT1 constructs. Stable expression of mCherry-KRAS4B was used as a plasma membrane marker. Representative images from one of three independent experiments (*n* = 3). **D**) Live-cell confocal images of HeLa cells transiently transfected with indicated mNeonGreen-RIT1 constructs. Representative images from two independent experiments (*n* = 2). For (C) and (D), scale bars = 15 µm.

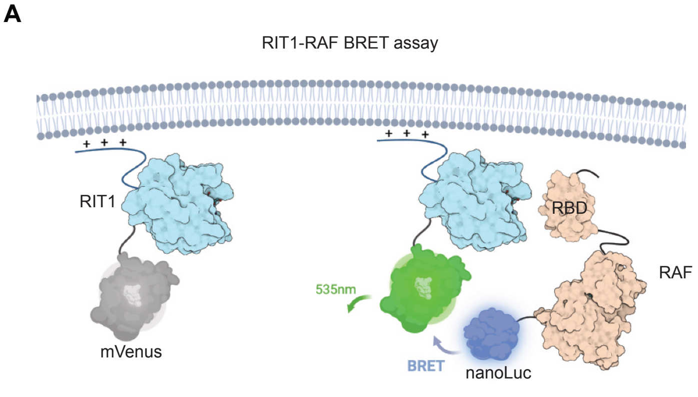

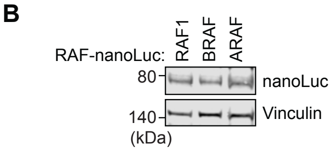

C

Interaction	$BRET_{50}$ Value
WT:RAF1	1.129 ±0.030
A57G:RAF1	0.110 ±0.019
WT: BRAF	1.510 ±0.108
A57G:BRAF	0.315 ±0.038
WT:ARAF	2.314 ±0.247
A57G:ARAF	0.968 ±0.066

fig. S3. RIT1 BRET assay reveals preferential binding to RAF1

(**A**) Schema of BRET assay designed to detect in vivo RIT1-RAF binding. (**B**) RAF-nanoLuc construct transfection was optimized to achieve comparable RAF expression levels. (**C**) $BRET_{50}$ values calculated from saturation curves in Fig. 3B.

A

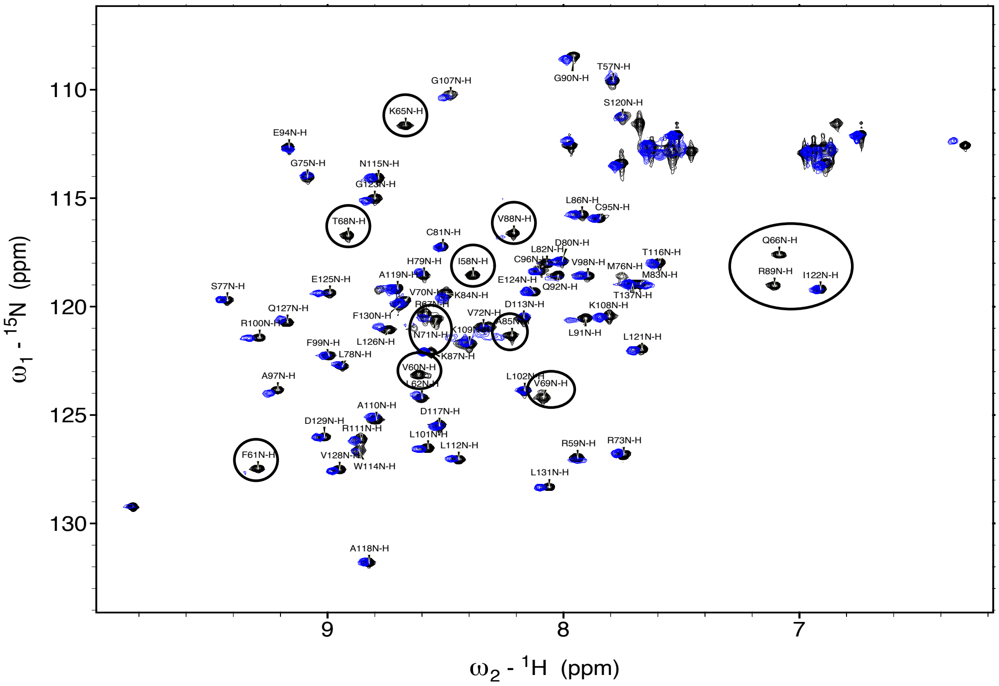

ω1 - 15N (ppm)
ω2 - 1H (ppm)
110
115
120
125
130
9
8
7
G90N-H
T57N-H
G107N-H
S120N-H
K65N-H
E94N-H
G75N-H
N115N-H
G123N-H
L86N-H
C95N-H
T68N-H
V88N-H
C81N-H
Q66N-H
I58N-H
D80N-H
L82N-H
T116N-H
H79N-H
C96N-H
V98N-H
M76N-H
M83N-H
R89N-H
I122N-H
A119N-H
E124N-H
S77N-H
E125N-H
Q92N-H
T137N-H
V70N-H
K84N-H
D113N-H
K108N-H
Q127N-H
F130N-H
V72N-H
R100N-H
N71N-H
A85N-H
L91N-H
L121N-H
F99N-H
L126N-H
K87N-H
L78N-H
V60N-H
L62N-H
L102N-H
V69N-H
A97N-H
A110N-H
D117N-H
D129N-H
R111N-H
L101N-H
L112N-H
R59N-H
R73N-H
F61N-H
V128N-H
W114N-H
L131N-H
A118N-H

B

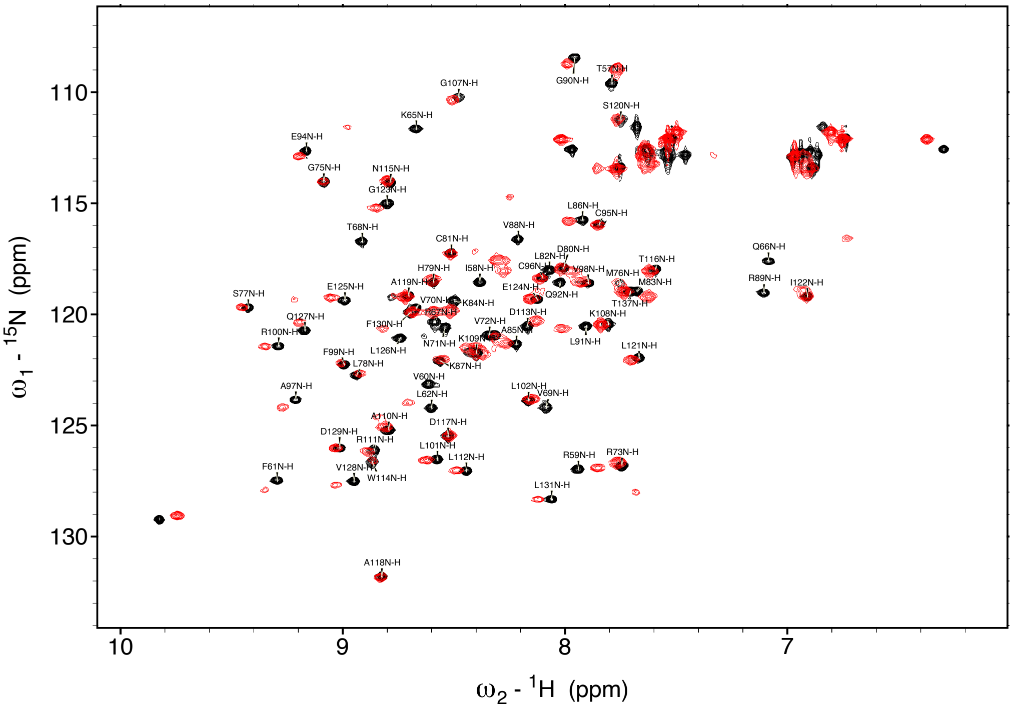

ω1 - 15N (ppm)
ω2 - 1H (ppm)
110
115
120
125
130
10
9
8
7
G90N-H
T57N-H
G107N-H
S120N-H
K65N-H
E94N-H
G75N-H
N115N-H
G123N-H
L86N-H
C95N-H
T68N-H
V88N-H
C81N-H
Q66N-H
L82N-H
D80N-H
T116N-H
H79N-H
I58N-H
C96N-H
V98N-H
M76N-H
R89N-H
M83N-H
I122N-H
S77N-H
E125N-H
A119N-H
E124N-H
Q92N-H
T137N-H
V70N-H
K84N-H
Q127N-H
D113N-H
K108N-H
F130N-H
V72N-H
R100N-H
A85N-H
L91N-H
N71N-H
L121N-H
F99N-H
L126N-H
K87N-H
L78N-H
V60N-H
A97N-H
L102N-H
L62N-H
V69N-H
A110N-H
D117N-H
D129N-H
R111N-H
L101N-H
R59N-H
R73N-H
L112N-H
F61N-H
V128N-H
W114N-H
L131N-H
A118N-H

C

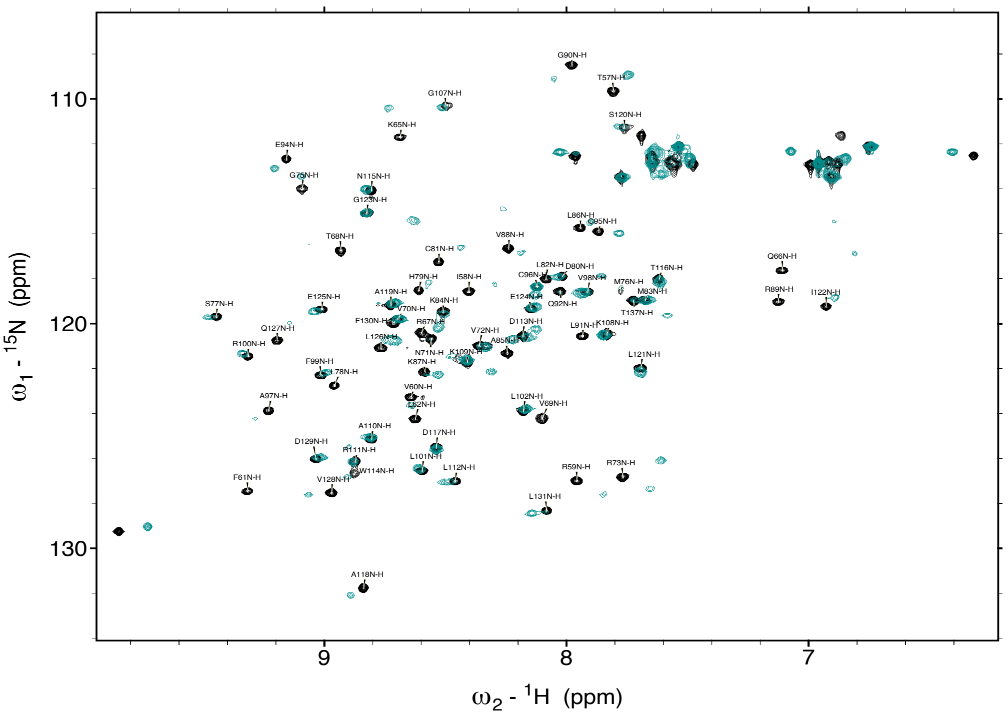

G90N-H
T57N-H
G107N-H
S120N-H
K65N-H
E94N-H
N115N-H
G75N-H
G123N-H
L86N-H
Q95N-H
T68N-H
V88N-H
C81N-H
L82N-H
D80N-H
T116N-H
Q66N-H
H79N-H
I58N-H
C96N-H
V98N-H
M76N-H
A119N-H
M83N-H
R89N-H
I122N-H
E125N-H
K84N-H
E124N-H
S77N-H
V70N-H
Q92N-H
T137N-H
F130N-H
R67N-H
D113N-H
L91N-H
K108N-H
Q127N-H
V72N-H
L126N-H
A85N-H
R100N-H
N71N-H
K109N-H
L121N-H
F99N-H
K87N-H
L78N-H
V60N-H
L102N-H
A97N-H
L62N-H
V69N-H
A110N-H
D117N-H
D129N-H
R111N-H
L101N-H
R73N-H
W114N-H
L112N-H
R59N-H
F61N-H
V128N-H
L131N-H
A118N-H
110
120
130
9
8
7
ω_1 - ^{15}N (ppm)
ω_2 - ^{1}H (ppm)

D

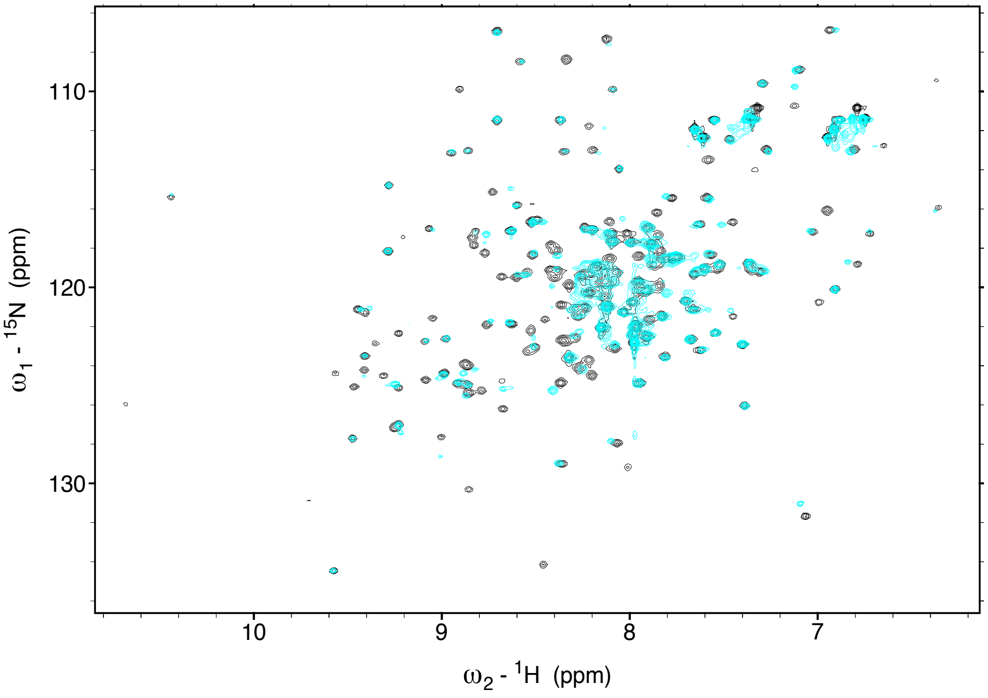

110
120
130
10
9
8
7
ω_1 - ^{15}N (ppm)
ω_2 - ^{1}H (ppm)

E

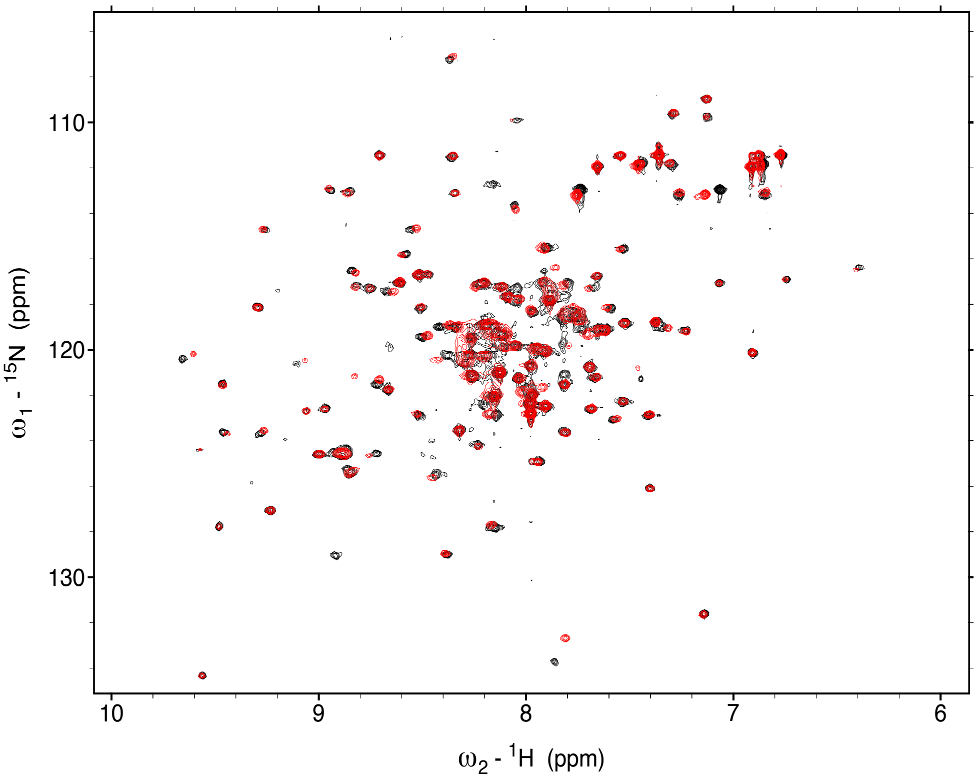

110
120
130
ω1 - 15N (ppm)
10
9
8
7
6
ω2 - 1H (ppm)

F

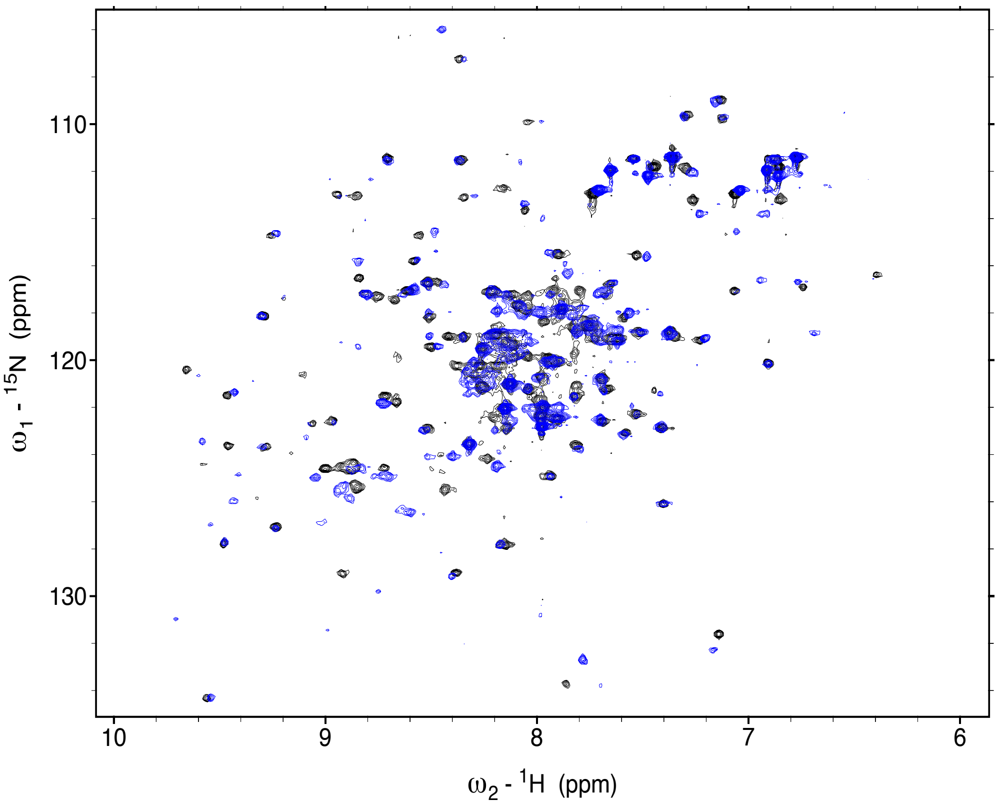

110
120
130
ω1 - 15N (ppm)
10
9
8
7
6
ω2 - 1H (ppm)

G

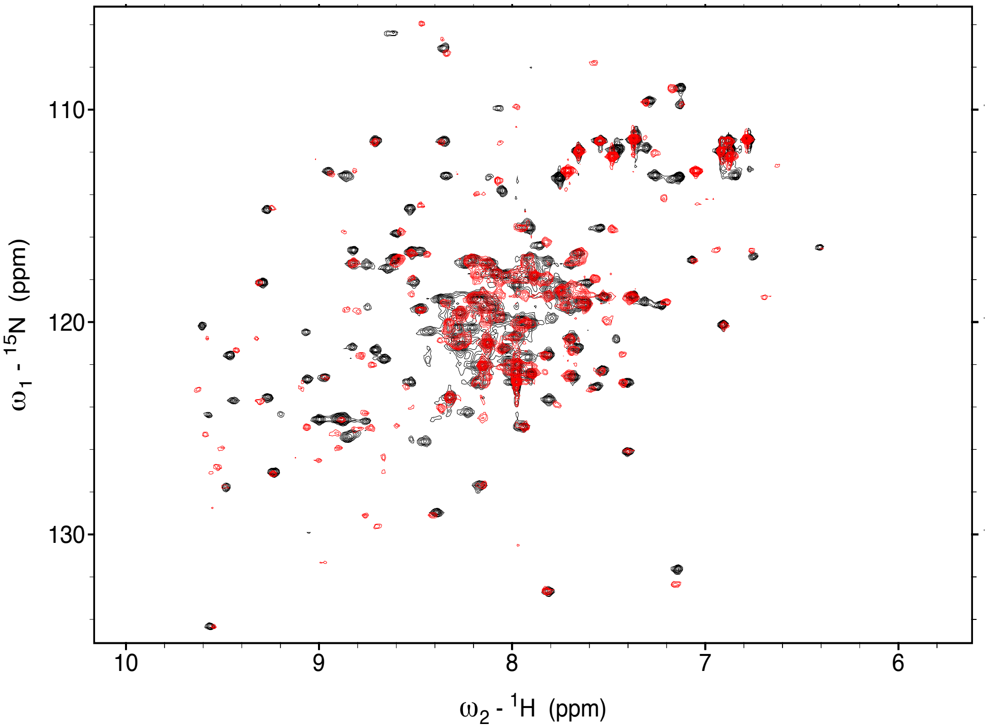

fig. S4. NMR spectra of RIT1-RAF1 RBD complexes

(**A**) Overlay ^{15}N -^{1}H HSQC spectra of ^{15}N RBD (black) and ^{15}N RBD in complex with unlabeled WT-RIT1-GppNHp (blue). (**B**) Overlay ^{15}N -^{1}H HSQC spectra of ^{15}N RBD (black) and ^{15}N RBD in complex with unlabeled RIT1^{A57G}-GppNHp (red). (**C**) Overlay ^{15}N -^{1}H HSQC spectra of ^{15}N RBD (black) and ^{15}N RBD in complex with unlabeled KRAS-GppNHp (cyan). (**D**) Overlay ^{15}N-^{1}H HSQC spectra of ^{15}N labeled WT-RIT1-GDP (black) with ^{15}N labeled RIT1^{A57G}-GDP (cyan). (**E**) Overlay ^{15}N-^{1}H HSQC spectra of ^{15}N labeled WT-RIT1-GppNHp (black) with ^{15}N labeled RIT1^{A57G}-GppNHp (red). (**F**) Overlay ^{15}N-^{1}H HSQC spectra of ^{15}N labeled RIT1-GppNHp in complex with RBD (blue) on ^{15}N labeled RIT1-GppNHp (black). (**G**) Overlay ^{15}N-^{1}H HSQC spectra of ^{15}N labeled RIT1^{A57G}-GppNHp in complex with RBD (red) on ^{15}N labeled RIT1^{A57G}-GppNHp (black).

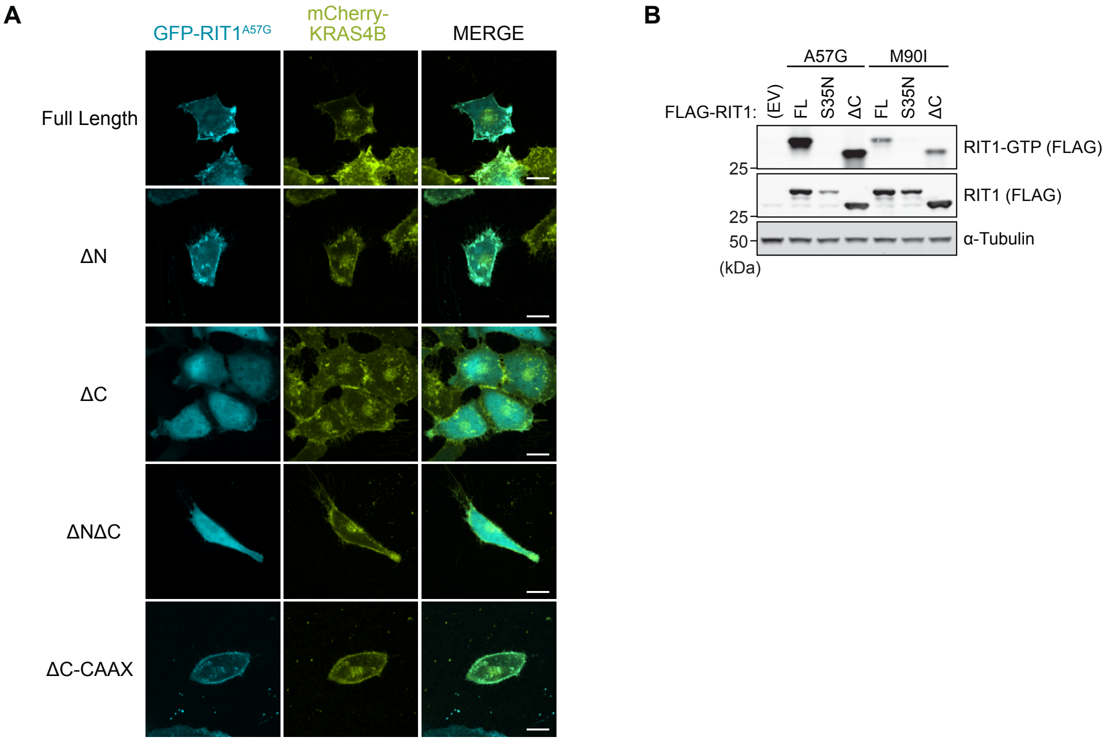

fig. S5. RIT1 C-terminus is essential for PM association but not GTP loading

(**A**) Live-cell confocal images of HeLa cells transiently transfected with indicated GFP-RIT1 constructs. Stable expression of mCherry-KRAS4B was used as a plasma membrane marker. Representative images from one of three independent experiments (n = 3). (**B**) Immunoblot analysis of indicated proteins from HEK-293 cells transiently transfected with indicated FLAG-tagged constructs and serum-starved for 16 h. GTP-bound RIT1 was precipitated with immobilized RAF1-RBD. One of two independent experiments is shown.

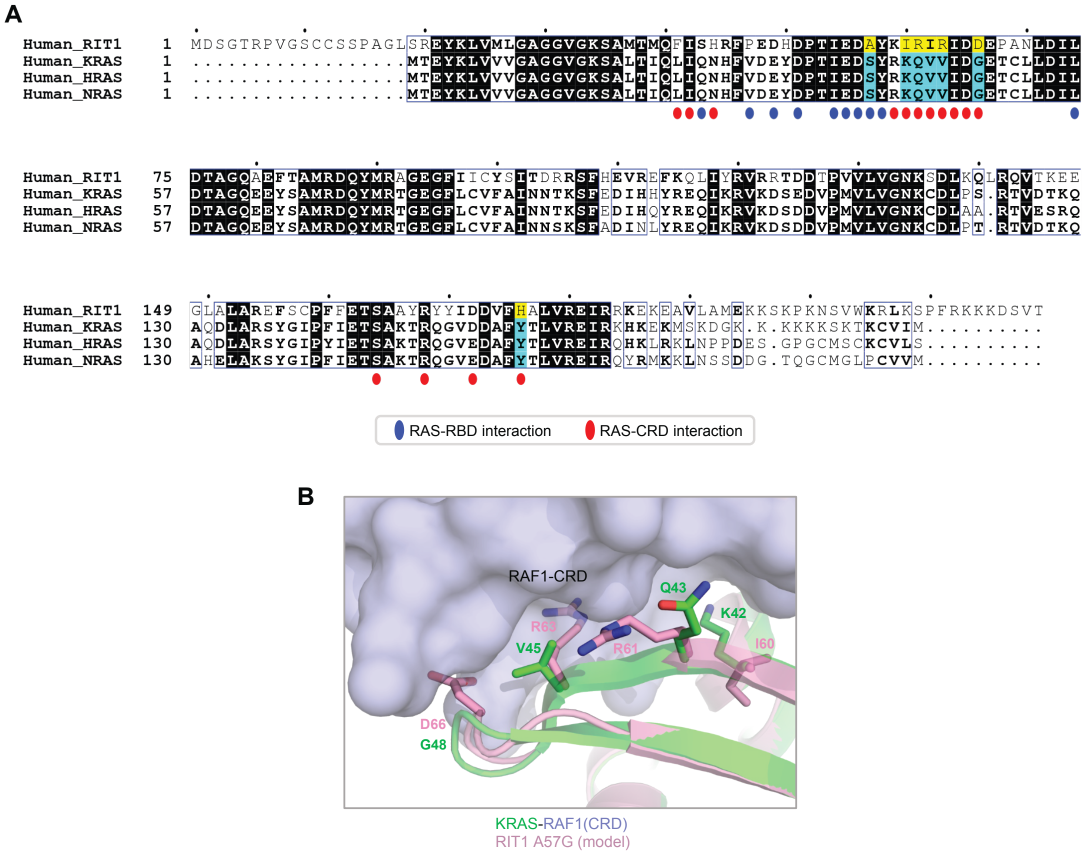

fig. S6. Analysis of RIT1 interaction with RAF1-CRD

(**A**) Sequence alignment of RIT1 with canonical RAS paralogs showing residues involved in interaction with RAF1 RBD-CRD. Totally conserved residues are bold and highlighted in black, while highly conserved residues are bold and highlighted in white. Non-conserved residues are only highlighted in white. Residues of K/N/H-RAS, which interact with RAF1 RBD and CRD, are denoted with blue stars and red ovals, respectively. Interswitch residues that play an important role in RAS-CRD interaction differ in RIT1 and RAS paralogs; these residues are highlighted in yellow (RIT1) and cyan (RAS). (**B**) The structural superposition of RIT1 with the KRAS-RAF1(RBD-CRD) complex shows that residues in the Interswitch region of KRAS and RIT1 that interact with CRD are different. Residues Q43, V45, and G48 in KRAS are replaced by R61, R63, and D66 residues in RIT1, and because of that, unlike RAS, RIT1 would not be able to form optimal interaction with RAF1-CRD.

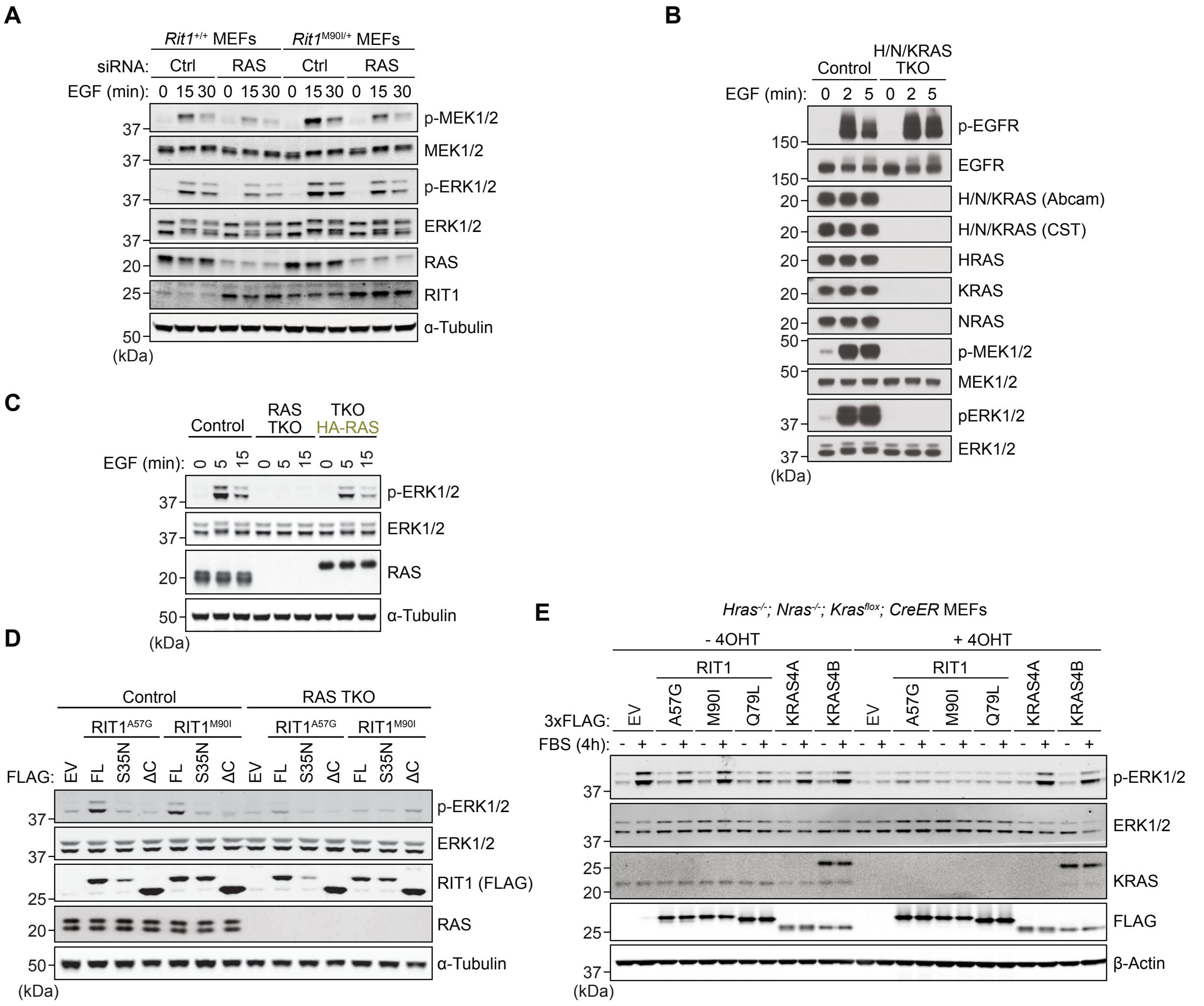

fig. S7. RIT1 protein stabilization fails to promote MAPK activation in the absence of RAS

(**A**) Immunoblot analysis of indicated proteins from primary MEFs that were serum-starved overnight and stimulated with 10 ng/ml EGF for indicated times, 72h post siRNA knockdown. One of three independent experiments is shown. (**B**) Immunoblot analysis of indicated proteins from Rasless (HRAS/NRAS/KRAS TKO) or control HEK-293 FlpIn cells serum-starved for 16 h and treated with 25 ng/ml EGF for indicated times. (**C**) Immunoblot analysis of indicated proteins from Rasless or control cells serum-starved for 16 h and treated with 10 ng/ml EGF for indicated times. Rasless cells were rescued with ectopic expression of HA-tagged HRAS, NRAS, KRAS4A, and KRAS4B (1:1:1:1 DNA ratio). One of two independent experiments is shown. (**D**) Immunoblot analysis of indicated proteins from Rasless or control cells transiently transfected with indicated FLAG-tagged RIT1 constructs or an empty vector (EV) control and serum-starved for 16 h. FL, full length. One of three independent experiments is shown. (**E**) Immunoblot analysis of indicated proteins from control (-4OHT) and Rasless (+4OHT) MEFs stably expressing indicated constructs, serum-starved overnight and stimulated with or without 10% FBS for 4 h. One of three independent experiments is shown.

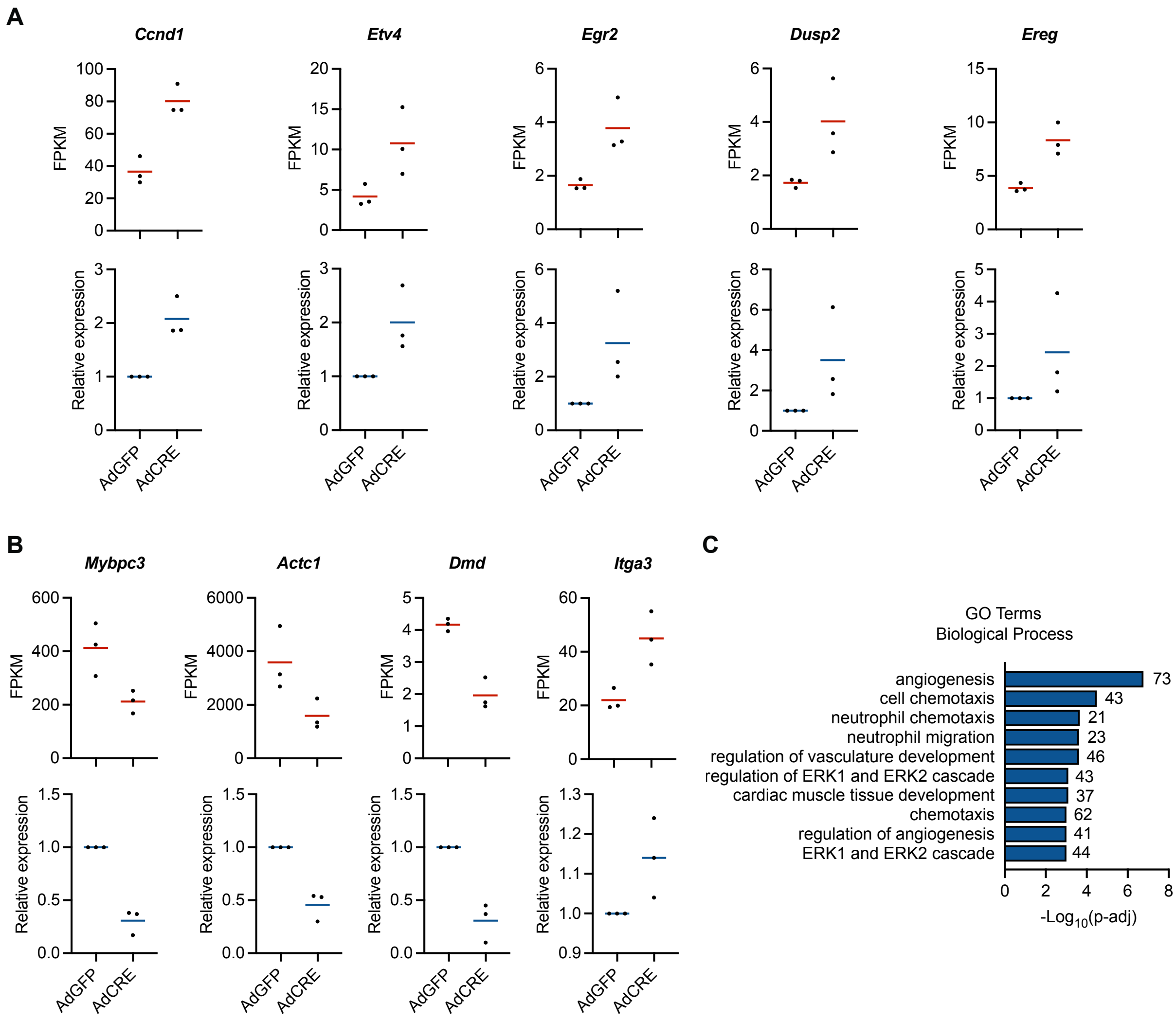

fig. S8. Analysis of gene expression elicited by RIT1^{M90I} expression or MEK inhibition

(**A**, **B**) Gene expression levels of indicated MAPK-regulated genes (A) or cardiomyopathy-associated genes (B) from primary *Rit1$^{LoxP\text{-}M90I}$* neonatal cardiomyocytes treated with adenovirus encoding Cre recombinase (AdCre) or GFP (AdGFP). Top panels display normalized mRNA transcript levels (FPKM) from RNA-seq transcriptomic profiling. Bottom panels display relative mRNA expression from an independent set of biological replicates by RT-qPCR. (**C**) Gene ontology (GO) enrichment analysis of genes downregulated in MEKi-treated hearts (MEKi vs. vehicle control) isolated from the murine preclinical trial described in Fig. 8D.

Supplementary Tables

Table S1. Broadened residues (underlined) and residues above 1.5σ reported in CSP plots

**^{15}N RBD<br>+ RIT1**	**^{15}N RBD<br>+ RIT1^{A57G}**	**^{15}N RBD<br>+ KRAS**	**^{15}N RIT1<br>+ RBD**	**^{15}N RIT1^{A57G}<br>+ RBD**	**^{15}N KRAS<br>+ RBD**
T57	T57	T57	R20	R20	Y4
<u>I58</u>	<u>I58</u>	I58	E21	<u>E21</u>	L6
<u>V60</u>	R59	R59	M26	M26	<u>V8</u>
F61	<u>V60</u>	V60	L27	<u>L27</u>	V9
L62	F61	<u>F61</u>	A29	G30	G15
<u>K65</u>	L62	<u>L62</u>	<u>H44</u>	<u>M37</u>	S17
<u>Q66</u>	<u>K65</u>	<u>K65</u>	A57	S43	<u>Q25</u>
<u>T68</u>	<u>Q66</u>	<u>Q66</u>	Y58	H44	H27
<u>V69</u>	R67	R67	I62	<u>K59</u>	<u>F28</u>
<u>N71</u>	<u>T68</u>	<u>T68</u>	A69	I62	V45
<u>V88</u>	<u>V69</u>	<u>V69</u>	N70	<u>A69</u>	<u>D47</u>
<u>R89</u>	<u>V70</u>	<u>V70</u>	L71	L71	<u>L56</u>
Q92	<u>N71</u>	R73	Q79	Q79	T74
A97	L82	G75	M85	A84	G75
L126	M83	<u>L78</u>	R86	M85	I84
Q127	K84	H79	E94	R86	<u>T87</u>
	<u>V88</u>	C81	G95	<u>E94</u>	<u>E91</u>
	<u>R89</u>	A85	F96	G95	Y96
	G90	K87	<u>S101</u>	F96	V103
	L91	V88	E110	Y100	D108
	Q92	<u>R89</u>	R112	S101	R123
	E94	G90	V121	V111	Y137
	A97	<u>L91</u>	R123	R112	R149
	D113	<u>Q92</u>	G133	Y119	F156
	E125	E94	S164	R123	Y157
	L126	A97	A166	<u>V132</u>	
	Q127	A118	Y170	G133	
	V128	I122	D172	S164	
	D129	L126	F175	R168	
		Q127	R180	Y170	
		V128	L191	D172	
		L131		R180	

Table S2. gRNA targeting sequences

Target	Sequence
HRAS	TTGGACATCCTGGATACCGC
KRAS	TTGGATATTCTCGACACAGC
NRAS	TTGGACATACTGGATACAGC
AAVS1	GGGACCACCTTATATTCCCA
Chromosome 3	GTTTAAAACTACCACTCCAC
Chromosome 15	GCCTATGGTCTGATAACAAT

Table S3. qPCR primers

Target	Forward primer (5'-3')	Reverse primer (5'-3')
Tbp	CCTTGTACCCTTCACCAATGAC	ACAGCCAACATTCACGGTAGA
Etv4	AGACTTCGCCTACGACTCA	CATAACCCATCACTCCATCACC
Ccnd1	CAACAACTTCCTCTCCTGCTAC	GCTTCAATCTGTTCCTGGCA
Dusp2	CGAGGGTTCCGATCTATGAC	CATTGAGAACTGCTGTGATGC
Ereg	CTTCTACAGGCAGTTATCAGCA	GTAGCCGTCCATGTCAGAAC
Erg2	TTGACCAGATGAACGGAGTG	GTGAAGGTCTGGTTTCTAGGTG
Dmd	GCTTATGTTGCCACCTCTGA	CTTCCGTCTCCATCAATGAACT
Itga3	CAGCATCCCTACCATCAACA	CCACAAGCACCAACCACA
Mybpc3	CTGGCAGAAGACTGTAACACA	TCAGTCTCACACAACAGCTTC
Actc1	CCAACCGTGAGAAGATGACC	TCGCCAGAATCCAGAACAATG
